# Estrogen Receptor-Beta2 (ERβ2)–Mutant p53–FOXM1 Axis: A Novel Driver of Proliferation, Chemoresistance, and Disease Progression in High Grade Serous Ovarian Cancer (HGSOC)

**DOI:** 10.3390/cancers14051120

**Published:** 2022-02-22

**Authors:** Chetan C. Oturkar, Nishant Gandhi, Pramod Rao, Kevin H. Eng, Austin Miller, Prashant K. Singh, Emese Zsiros, Kunle O. Odunsi, Gokul M. Das

**Affiliations:** 1 Department of Pharmacology and Therapeutics, Roswell Park Comprehensive Cancer Center, Buffalo, NY 14263, USA; chetan.oturkar@roswellpark.org (C.C.O.); ngandhi@carisls.com (N.G.); pramodra@buffalo.edu (P.R.); 2 Department of Biostatistics & Bioinformatics, Roswell Park Comprehensive Cancer Center, Buffalo, NY 14263, USA; kevin.eng@roswellpark.org (K.H.E.); austin.miller@roswellpark.org (A.M.); 3 Genomic Shared Resource, Roswell Park Comprehensive Cancer Center, Buffalo, NY 14263, USA; prashant.singh@roswellpark.org; 4 Department of Gynecologic Oncology, Roswell Park Comprehensive Cancer Center, Buffalo, NY 14263, USA; emese.zsiros@roswellpark.org (E.Z.); odunsia@bsd.uchicago.edu (K.O.O.)

**Keywords:** high grade serous ovarian cancer, estrogen receptor-beta2, mutant p53, FOXM1, therapeutic resistance, carboplatin, patient tumors, proximity ligation assay, apoptosis, cell proliferation

## Abstract

**Simple Summary:**

High grade serous ovarian cancer (HGSOC) is the most common and lethal subtype of ovarian cancer without effective therapeutic options. The high prevalence of mutations (~96%) in tumor suppressor p53 is a hallmark of HGSOC. Estrogen receptor-beta (ERβ) has been reported to be another important player in HGSOC, although the pro-versus anti-tumorigenic role of its different isoforms remains unclear. The aim of this study was to analyze the crosstalk between ERβ and mutant p53 and its impact on the pro-tumorigenic processes in HGSOC. Using the HGSOC cell line models and patient tumor tissue specimens, we demonstrated functional interaction between the ERβ2 isoform and mutant p53 and their ability to co-dependently increase *FOXM1* gene transcription, decrease cell death, increase cell proliferation, and mediate resistance to carboplatin treatment. Furthermore, high levels of ERβ2 as well as FOXM1 correlated with worse patient survival. Collectively, our data suggest that the ERβ2-mutant p53-FOXM1 axis could be a novel therapeutic target for HGSOC.

**Abstract:**

High grade serous ovarian cancer (HGSOC) is the most common and lethal subtype of epithelial ovarian cancer. Prevalence (~96%) of mutant p53 is a hallmark of HGSOC. Estrogen receptor-beta (ERβ) has been reported to be another important player in HGSOC, although the pro-versus anti-tumorigenic role of its different isoforms remains unsettled. However, whether there is functional interaction between ERβ and mutant p53 in HGSOC is unknown. ERβ1 and ERβ2 mRNA and protein analysis in HGSOC cell lines demonstrated that ERβ2 is the predominant isoform in HGSOC. Specificity of ERβ2 antibody was ascertained using cells depleted of ERβ2 and ERβ1 separately with isoform-specific siRNAs. ERβ2-mutant p53 interaction in cell lines was confirmed by co-immunoprecipitation and in situ proximity ligation assay (PLA). Expression levels of ERβ2, ERα, p53, and FOXM1 proteins and ERβ2-mutant p53 interaction in patient tumors were determined by immunohistochemistry (IHC) and PLA, respectively. ERβ2 levels correlate positively with FOXM1 levels and negatively with progression-free survival (PFS) and overall survival (OS). Quantitative chromatin immunoprecipitation (qChIP) and mRNA expression analysis revealed that ERβ2 and mutant p53 co-dependently regulated *FOXM1* gene transcription. The combination of ERβ2-specific siRNA and PRIMA-1^MET^ that converts mutant p53 to wild type conformation increased apoptosis. Our work provides the first evidence for a novel ERβ2-mutant p53-FOXM1 axis that can be exploited for new therapeutic strategies against HGSOC.

## 1. Introduction

Epithelial ovarian cancer (EOC) is a leading cause of fatality among gynecological cancers [1]. It represents a group of complex and heterogeneous diseases of which high-grade serous ovarian cancer (HGSOC) is the most aggressive and accounts for more than 60% of EOCs and over 70% of all deaths due to ovarian cancer [2]. HGSOC is commonly diagnosed at an advanced stage [3]. Peritoneal metastasis is the main reason for the poor prognosis of therapy–resistant HGSOC. Recent studies have indicated that HGSOC may have dual origins from both fallopian tube and ovarian surface epithelium [4,5]. HGSOCs are typically high-grade, genetically unstable, and harbor p53 (*TP53*) mutations [2,6]. While wild type p53 is expressed in low-grade serous carcinomas (LGSOC), mutant or null alleles of p53 are universally (~96%) present in HGSOC [2]. A study based on the International Agency for Cancer Research (IARC) p53 database reported that over 70% of p53 mutations were missense mutations (which causes amino-acid substitution) with smaller contributions from frameshift, nonsense, and splice mutations [7]. Individual hotspot mutations could have a different impact on HGSOC patient outcomes [8]. Mutant p53 was reported to promote epithelial ovarian cancer by regulating tumor differentiation, metastasis, and responsiveness to steroid hormones [9]. Based on TCGA analyses, HGSOC treated with standard platinum- and taxane-based chemotherapy had higher rates of resistance in tumors with oncomorphic p53 mutations with predicted gain-of-function (GOF) effects in the absence of the wild type *TP53* allele [10,11,12]. Importantly, while many driver mutations evolved in response to therapeutic intervention contributing to resistance, p53 mutations are unchanged during the course of the disease [7,13,14].

Various research groups have reported on the roles of estrogen signaling in ovarian cancer [15]. Estrogen action is mediated by two receptors: estrogen receptor-alpha (ERα/ESR1) and estrogen receptor-beta (ERβ/ESR2), transcriptional regulators belonging to the large family of nuclear receptors. Although expression of ERα in HGSOC has been reported [16,17,18] and was shown to be positively correlated with lymphovascular space invasion (LVSI), a prognostic indicator of poor survival [19], a large study conducted by the Ovarian Tumor Tissue Analysis consortium in 1742 HGSOC cases showed that ERα was not associated with improved HGSOC survival [18]. Although there are various reports on the role of ERβ in HGSOC, they have been inconsistent [20,21]. While some studies have reported ERβ to have anti-proliferative effects in tumor cell lines and cell line-derived xenografts [20,22,23,24,25,26] and to be associated with longer progression-free survival (PFS) and/or overall survival (OS) of patients [27,28,29], others have reported pro-tumorigenic effects [20,30,31]. Differences in cell lines, patient cohorts, antibodies, immunohistochemistry (IHC), and protocols used in the studies could have contributed to the inconsistencies besides the differences in the isoforms of ERβ that were analyzed. Estrogen signaling interacts with various proteins and signaling pathways. Although the unfavorable prognostic role of ERβ2 in HGSCO [20] and mitochondrial ERβ2 driving antiapoptotic pathways have been reported, interaction between ERβ2 and p53 signaling remains unknown. Because of the absence of wild type p53, a transcriptional repressor of *FOXM1* [32,33], the latter is highly expressed in HGSOC and is a major driver of these cancers [2,34]. Although interaction between ERβ and p53 has been reported in other cancers [35,36,37], whether they interact in HGSOC and drive oncogenesis via FOXM1 remained unknown until we showed that such interaction with functional consequences occurs in HGSOC [38]. Identifying and understanding molecular mechanisms of signaling pathways that crosstalk to drive ovarian cancer and contribute to therapeutic resistance is necessary to gain insight to develop novel therapeutic strategies. Using multiple HGSOC cell lines authenticated by genomic profiling [39] and in-house HGSOC patient tumor tissues, we addressed ERβ2-p53 crosstalk and its functional implications in tumor progression and therapeutic resistance.

## 2. Materials and Methods

### 2.1. Cell Culture and Reagents

OVCAR3 (HGSOC cell line expressing mutant p53 R248Q), A2780 (none-serous ovarian cancer cell line expresses expressing wild type p53), KURAMOCHI (HGSOC cell line expressing mutant p53 D281Y) and OV-90 (HGSOC cell line expressing mutant p53 S215R), and MDA-MB-231 (TNBC cell line expressing mutant p53 R280K) were cultured in Dulbecco’s Modified Eagle’s medium (DMEM) (Corning, Corning, NY, USA, Cat #10-013-CM) supplemented with 10% fetal bovine serum (FBS) (GIBCO/Thermo Fisher, Waltham, MA, USA, Cat #20937-028), penicillin and streptomycin (50 g/mL). All cell lines were grown at 37 °C, under 5% CO_2_, in a humidified incubator. FT282-C11 cell line (immortalized/untransformed fallopian tube fimbrial epithelial cells expressing mutant p53 R175H) was maintained in DMEM/F12 without L-Glutamine (Invitrogen/Thermo Fisher, Waltham, MA, USA, Cat #21331-020) medium containing 10% Horse Serum (Gibco/Thermo Fisher, Waltham, MA, USA, Cat #16050130). FT282-C11 cells were provided by Dr. Michael Higgins (Roswell Park Comprehensive Cancer Center/RPCCC). Kuramochi cells were provided by Dr. Adam Karpf (University of Nebraska, USA). OV-90 cells were purchased from ATCC. All cell lines were authenticated by short tandem repeat (STR) analysis. Carboplatin (Cat #41575-94-4, Millipore Sigma-Calbiochem, St. Louis, MO, USA) was provided by Dr. Xinjiang Wang (RPCCC). PRIMA-1^MET^ (Cat #3710) was purchased from TOCRIS Bio-Techne Corporation, Minneapolis, MN, USA. DPN Cat #1494), β-Estradiol (Cat #E2758, Sigma-Aldrich, St. Louis, MO, USA), PHTPP (Cat #2662), and 4-Hyrdoxy tamoxifen (Cat #3421) from TOCRIS, Bioscience, Bristol, UK).

### 2.2. siRNAs and Transfection

Cells were seeded and maintained in their respective media for 24 h. Non-specific stealth control siRNNA (Invitrogen/Thermo Fisher, Waltham, MA, USA, Cat #12935-300), or siRNAs against ERβ1 and ERβ2 sequences were transiently transfected using Lipofectamine 3000 (Thermo Fisher Scientific, Waltham, MA, USA, Cat #l3000015), as per the manufacturer’s protocol. Cells were transfected with a final concentration of 120 nM stealth control siRNA (si-NS), si-ERβ1 or si-ERβ2 for 48 h or 50 nM p53 siRNA for 24 h. Post transfection, cells were harvested for downstream analysis. Sequence information of siRNAs is provided in Table S1.

### 2.3. RNA Isolation and Quantitative Real Time PCR (RT-qPCR) Assays

Total cellular RNA was isolated using TRizol reagent (Invitrogen, Waltham, MA, USA, Cat #15596026) as per the manufacturer’s protocol; RNA Integrity and yield was checked on a 1.5% agarose gel and quantified using a Nanodrop 8000 spectrophotometer. Isolated RNA treated with DNAse I, amplification grade I (Invitrogen/Thermo Fisher, Waltham, MA, USA) was used for cDNA synthesis. The first strand cDNA pool was synthesized using 1 µg of total RNA and BioRad’s iScript™ cDNA synthesis kit (BioRad, Hercules, CA, USA), Cat #170-8891) in a 20 µL reaction volume. Quantitative real time (qRT-qPCR) was performed using an iTaq Universal SYBR Green supermix (BioRad, Hercules, CA, USA, 1725121) in a 10 μL reaction in Applied Biosystem’s ABI Prism 7300 Real time PCR machine: 50 °C for 15 s (1 cycle); 95 °C for 10 min (1 cycle); 95 °C for 15 s, followed by 60 °C for 45 s (40 cycles). Dissociation curves were used to confirm the detection of a single amplicon. Data acquisition and analysis was carried out by ABI’s 7300 system sequence detection software V1.4. The relative target levels were determined by the ΔΔCT method using β-actin mRNA as reference gene endogenous controls. All experimental and control groups were performed in biological and technical triplicates. Details of PCR primers are shown in Table S1.

### 2.4. Immunoblotting

For immunoblotting, cells were scraped in ice cold 1xPBS, followed by centrifugation at 5000 rpm for 5 min at 4 °C. The cell pellet was lysed in RIPA buffer and quantification of protein was performed using Bradford reagent (BioRad, USA Cat #5000006) as per the manufacturer’s protocol. The protein lysates were subjected to SDS-PAGE, followed by immunoblotting. The details of the antibodies along with dilutions used are provided in Table S2. The specificity of the ERβ2 antibody was ascertained by knocking down ERβ2 with specific siRNA and the overexpression of the ERβ2 expression plasmid (see Figure S3).

### 2.5. Co-Immunoprecipitation (Co-IP) Assay

OVCAR3 cells were harvested and lysed in an ice cold RIPA lysis buffer (50 mM Tris-HCl pH 8.0, 1% NP-40, 150 mM NaCl, 0.5% deoxycholate, 0.1% SDS, 5 mM EDTA) and EDTA-free “Roche cOmplete” protease inhibitor cocktail (Millipore Sigma, St. Louis, MO, USA, Cat #11873580001) for 30 min. Cell lysates were further centrifuged at 15,000 rpm for 10 min at 4 °C and then pre-cleared with protein G agarose (Life Technologies/Thermo Fisher, Waltham, MA, USA, Cat #15920010) for 1 h at 4 °C. Approximately 2.5 mg of precleared lysate was incubated with 5 mg of ERβ2 or IgG specific antibody overnight. After 24 h, immunogen-antibody complexes were incubated with freshly prepared a lysis buffer saturated with protein G agarose beads at 4 °C. After 3 h, the beads were then washed three times with wash buffer (RIPA buffer without SDS and Triton). Protein complexes were eluted in 2× sample buffer (100 mM Tris, pH 6.8; 4% SDS; 20% glycerol; 10% 2-mercaptoethanol; 0.2% bromophenol blue) and processed for immunoblotting. ERβ2 and IgG antibodies are detailed in Table S2. The specificity of the ERβ2 antibody was ascertained using cells where ERβ2 and ERβ1were depleted separately with isoform-specific siRNAs.

### 2.6. Chromatin Immunoprecipitation (ChIP) Assay

OVCAR3 (1 × 10^7^) cells were washed with ice cold 1XPBS and incubated for crosslinking with 1% formaldehyde solution (Sigma, St Louis, MO, USA, Cat #F1635). The crosslinking reaction was stopped by adding 0.125 M Glycine (VWR, Atlanta, GA, USA, Cat # 56-40-6). Cells were washed three times and scrapped in Szak’s RIPA buffer (150 mM NaCl, 1.0% NP-40, 0.5% deoxycholate, 0.1% SDS, 50 mM Tris-HCl pH 8, 5 mM EDTA and 0.5 mM PMSF free Complete protease inhibitor cocktail (Millipore Sigma, St. Louis, MO, USA, Cat #11873580001). After incubating the cell pellet with the lysis buffer for 10 min on ice, the cell lysate was sonicated using Diagenode Biorupter Sonicator (Diagenode, NJ, USA, Cat #B01010002) at the setting “HIGH” with 30 sec ON/30 s OFF phases for 5-min interval cycle. The sample was sonicated to obtain optimum smear of approximately 100–500 bps. Approximately 1 mL cell lysate was incubated with ERβ2 specific or IgG (negative control) specific antibody overnight on rotor at 4 °C, followed by further incubation at 4 °C for 2 h after addition of 20 µL Pierce Protein A/G magnetic beads (Thermo Fisher, Waltham, MA, USA, Cat #88802). Antibody-chromatin complex linked magnetic beads were separated using a magnetic rack and washed (5 min each on rocker) with series of buffers (low salt immune complex, high salt immune complex wash buffer, LiCl immune complex wash buffer, 2× TE Buffer). After the final wash, 250 mL of 1.5× Talianidis elution buffer (70 mM Tris-HCl pH 8, 1 mM EDTA and 1.5% SDS) was used to separate immunocomplex from beads. Eluted immunocomplex was incubated further for 10 min at 65 °C, followed by boiling at 95 °C for a few seconds. Reverse crosslinking was performed by adding 13 mL of 4 M NaCl to each sample and further incubation at 65 °C for 5 h. After 24 h, sample was incubated with 2 mL of 2 mg/mL Proteinase K (New England Biolabs, Ipswich, MA, USA, Cat #P8102S) at 45 °C for 1 h. DNA was precipitated using Phenol-chloroform extraction followed by ethanol precipitation. Purified DNA was resuspended in 50 mL 1× E buffer and analyzed with quantitative real-time PCR (qRT-PCR) using nonspecific primers (region −2792 bp to −2965 bp) and estrogen receptor response element (ERE) site primers (−2565 bp to −2965 bp) on the *FOXM1* gene promoter. Sequence details of the ChIP primers are provided in the Table S1.

### 2.7. Flow Cytometry Analysis

Cells were transfected with siRNA for 48 h, followed by treatment with carboplatin (4 µM) for 24 h. Post treatment, cells were washed with PBS followed by Annexin V (FITC) and PI staining as per manufacturer protocol (BD Bioscience, Franklin Lakes, NJ, USA, Cat #556570). Stained cells were analyzed by FACS Calibur flow cytometer and apoptotic data was plotted using Winlist 8.0 software. The assay was performed in triplicates and statistical tests were computed using the ANOVA test.

### 2.8. Colony Formation Assay

48 h post siRNA transfection and drug treatment, approximately 2000 cells/cm^2^ were seeded and allowed to grow for 8–10 days. The cells were fixed with 10% formaldehyde solution for 15 min at room temperature. Subsequently, cells were stained with 0.1% crystal violet solution for 15 min at room temperature. Unabsorbed crystal violet dye was washed away by submerging the plate several times in running water. Plates were completely air dried at ambient temperature for several hours and photographed. For quantitation purposes, the absorbed dye, which is proportional to the cell mass, was extracted with 10% acetic acid solution. 100 µL of extract per sample was loaded into 96-well plates, and the absorbance at 595 nm was measured using a Synergy 2 (BioTek Winooski, VT, USA) plate reader.

### 2.9. Immunohistochemistry (IHC) on Patient Tissue Microarray (TMA)

#### 2.9.1. Patient Characteristics and Tissue Procurement

Primary and metastatic tumors from 44 patients were used to create the TMA. All patients had high grade serous ovarian or primary peritoneal cancer. The median age of the patients was 63 years (range 39–88). Ninety-eight percent of the patients were Caucasian and 2% were American Indian. All patients underwent primary debulking surgery without receiving neoadjuvant chemotherapy. Tumor stage distribution was stage 1A (2%), stage 2B (2%), stage 3 (60%), stage 4 (27%), and undefined (9%).

All activities of tissue procurement are directed and recorded by the Laboratory Information Management System (LIMS) at RPCCC. LIMS associates data to a sample family, including where/how the specimen was obtained, what precisely the sample is, and what the quality of that sample is. The system also tracks the life history of a biospecimen throughout its procurement, storage and distribution along with all associated core laboratory data.

#### 2.9.2. TMA Construction

Two ovarian TMAs (GynCa10 and GynCa11) constructed from primary tumors and their counterpart peritoneal metastatic tumor tissues were made available by the RPCCC Pathology Network Shared Resource (PNSR). Three 1-mm tissue cores from each formalin-fixed paraffin embedded (FFPE) donor blocks were precisely arrayed onto a new recipient paraffin block that included tumor specimens and controls. Eligible patients had surgeries performed between 1995 and 2008 at RPCCC, Buffalo, NY. Specimens for controls within the TMA consisted of multiple cores of normal tissue from 10 different organs including heart, colon, kidney, adrenal, ovary, myometrium, brain, thyroid, lung, and prostate, thereby representing more than 20% of all the cores in a TMA.

The usage of TMAs with patient tumor tissues was approved (BDR 060615) by the Institutional Review Board (IRB) for all ethical compliances consistent with federal, state, and local requirements. All tumor tissue specimens were obtained in de-identified format.

#### 2.9.3. Antigen Retrieval

FFPE sections from GynCa10 and GynCa11 TMAs together contained 132 tumor tissues, of which 44 were primary tumor tissues, along with their respective peritoneal metastatic tumor tissues; 44 additional cases were primary tumors, and another 10 were metastatic tumors. Formalin-fixed paraffin sections of these TMAs were cut at 4 µM, placed on charged slides, and dried at 60 °C for one hour. Slides were cooled to room temperature and added to the Dako Omnis autostainer, where they were deparaffinized with Clearify (American Mastertech, McKinney, TX, USA, Cat # Caclegal) and rinsed in water. Flex TRS High pH (Dako, Carpinteria, CA, USA, Cat # GV804) was used for target retrieval for 30 min for FOXM1 and p53. TRS Low pH (Dako, cat# GV805,) was used for target retrieval for ERβ2. Slides were incubated with FOXM1 antibody (sc-502, Santa Cruz Biotechnology, Dallas, TX, USA) or p53 antibody (Santa Cruz Biotechnology, Dallas, TX, USA, Cat # sc-126) for 30 min at 1:50 dilution, followed by Flex HRP polymer (Dako, Cat # DM843) which was applied for 30 min. For staining slides for ERβ2, the ERβ2 antibody (BioRad, Hercules, CA, USA, Cat # MCA2279,) was applied at 1:200 dilution for 20 min followed by Flex HRP polymer for 20 min. DAB (Diaminobenzidine) was applied (Dako, Cat # K3468) for 5 min for visualization. Slides were counterstained with Hematoxylin for 8 min and then put into water. After removing slides from the Omnis, they were dehydrated, cleared, and coverslips were mounted on to them.

#### 2.9.4. Quantitative Scoring of Nuclear ERβ2 and TP53 and FOXM1 Immunohistochemistry Signals

TMA slides were digitally scanned using an Aperio Scanscope (Aperio Technologies, Inc., Vista, CA, USA) with 20× bright-field microscopy. These images were accessible using Spectrum (Aperio Technologies, Inc., Vista, CA, USA), a web-based digital pathology information management system.

Once the slides were scanned, the Aperio ImageScope version 12.4.3.7009 (Aperio Technologies, Inc., Vista, CA, USA) was used to view images for analysis. An annotation layer was created for each core of interest in the TMA for targeting cells of interest for analysis. Regions were identified and annotated to appropriately represent the heterogeneity of staining of each TMA core and to reduce irrelevant regions from image analysis calculations.

The Aperio platform was used to develop quantitative image analysis algorithms for the quantification of slides. Briefly, these algorithms use color de-convolution to separate diaminobenzidine (DAB) from the haematoxylin counterstain, thereby providing stain separation. Each algorithm is tailored to fine tune the cell feature detection using cellular, nuclear, and stain parameters, creating a specific macro based on the cell compartment location of each target protein. The algorithm was adjusted for each antibody target and tissue combination to optimize results.

The Cytoplasmic algorithm was used to create a macro for both ERβ2 and FOXM1. The macro analyzes DAB staining intensity and the percentage of cells containing the stain within the cytoplasm compartment. The analysis results provide the total number of cells, percentage per scoring class, and H- Score. The H-score is a weighted index score derived from the average intensity of the staining of the cytoplasm according to the threshold intervals set in the algorithm. This score equals = 1× (%1+) + 2× (%2+) + 3× (%3+), with the score ranging between 0 and 300, where 300 represents 100% of cells being 3+.

The Nuclear algorithm was used to create a macro for both ERβ2 and p53. The macro detects the positive (DAB) nuclear staining for the individual tumor cells and quantifies their staining intensity. The analysis results provide the total number of detected cells, the percentage of cells per scoring class (0, 1+, 2+ and 3+) and the percentage of positively stained cells along with each sample’s average staining intensity of the positive nuclei as a score of 0, 1+, 2+ and 3+. The H-score was manually generated based on the same formula above.

### 2.10. Proximity Ligation Assay (PLA)

#### 2.10.1. Fluorescence-Based PLA

In situ PLA in cultured cells was carried out using the Duolink II reagent kit (Millipore-Sigma, St Louis, MO, USA cat# DUO92008) and supplier’s protocol. Briefly, 10,000 cells were seeded on 12 mm coverslips (Thermo Fisher Scientific, Grand Island, NY, USA) in 24-well plates. After 12–24 h, cells were fixed with freshly prepared 2% paraformaldehyde (Millipore-Sigma, St Louis, MO, USA cat#P6148) solution in PBS, pH 7.4 at room temperature for 20 min. Subsequently, the coverslips were washed twice with 1 mL of PBS, blocked and permeabilized with a solution containing 2% BSA and 0.1% Triton-X-100 in PBS, pH 7.4, for 1 h at room temperature, followed by nuclear permeabilization using a buffer containing 1% BSA and 0.1% NP40 in PBS, pH 7.4, for 15 min at room temperature. Mouse and rabbit primary antibodies were diluted appropriately in antibody dilution buffer (supplied in the kit) and were applied to the coverslips in an open droplet manner and incubated at room temperature for 1 h in a humidified chamber. The remainder of the protocol, which included secondary probe hybridization, ligation, and amplification, were carried out as per the manufacturer’s instructions. Coverslips were mounted with the supplied mounting media containing DAPI. Photographs were taken with an AXIOSKOP (Carl Zeiss, Jena, Germany) fluorescent microscope fitted with a Hamamatsu 3CCD digital camera and ImagePro Plus Software. PLA signals were quantitated by ImageJ software (NIH, Bethesda, MD, USA) or by manual counting.

#### 2.10.2. Bright-Field PLA

FFPE ovarian TMAs (GynCa10 and GynCa11) were deparaffinized in xylene for 3 min and hydrated through an ethanol gradient (3 min in 100%, 3 min in 70%). After hydration, TMAs were washed twice with tris-buffered saline with Tween 20 (TBST) followed by antigen retrieval using antigen retrieval buffer (EnVision FLEX Target Retrieval Solution, High pH 50× (Dako Omnis, Cat # GV804) in a steamer at 100 °C for 1 h. TMA slides were cooled at room temperature and further quenched by Duolink hydrogen peroxide (Millipore-Sigma, St Louis, MO, USA, Cat # DUO82054) for 10 min followed by blocking by Duolink blocking solution (Millipore-Sigma, Cat # DUO82007) for 5 min. Primary antibodies of ERβ (Santa Cruz Biotechnology, Dallas, TX, USA, Cat # 14C8) (1:100 dilution in antibody dilution buffer) and FL393 (Santa Cruz Biotechnology, Cat # DO1(1:100 dilution in antibody dilution buffer) were applied onto each TMA (50 μL) and incubated further for 1 h in a humidified chamber. Following the primary antibody incubation, mouse PLUS and rabbit MINUS secondary PLA antibodies were diluted appropriately in an antibody dilution buffer and were applied to the TMA (50 μL) and incubated for 1 h in a humidified chamber. The ligation and amplification reactions were carried out on all slides as described by the manufacturer (Millipore-Sigma, St. Louis, USA, Cat # DUO92012A). After amplification, TMAs were incubated with a Horseradish peroxidase (HRP)-labeled hybridization probe for 1 h in a humidity chamber at 37 °C followed by incubation with substates (A, B, C and D) as per the manufacturer’s protocol (Millipore-Sigma, St. Louis, USA, Cat # DUO92012B). Following two washes with distilled water, TMAs were then counterstained for 1 min in the Duolink nuclear stain (Millipore-Sigma, St. Louis, USA, Cat # DUO82059). TMA sections were dehydrated (2 × 3 min in 95% ethanol; 2 × 1 min in 100% ethanol) and transferred to xylene (3 min) and cover slips were mounted using mounting media (Thermo Fisher Scientific, Grand Island, NY, USA, Cat # 23425401). After overnight incubation, TMAs were scanned by Leica Aperio ScanScope XT and images were captured using the Aperio Spectrum Digital Slide System. ERβ2-p53 interaction signal dots per 300 nuclei were manually counted in all tumor tissues on the TMAs.

### 2.11. Statistical Analysis

**Figure 1:**
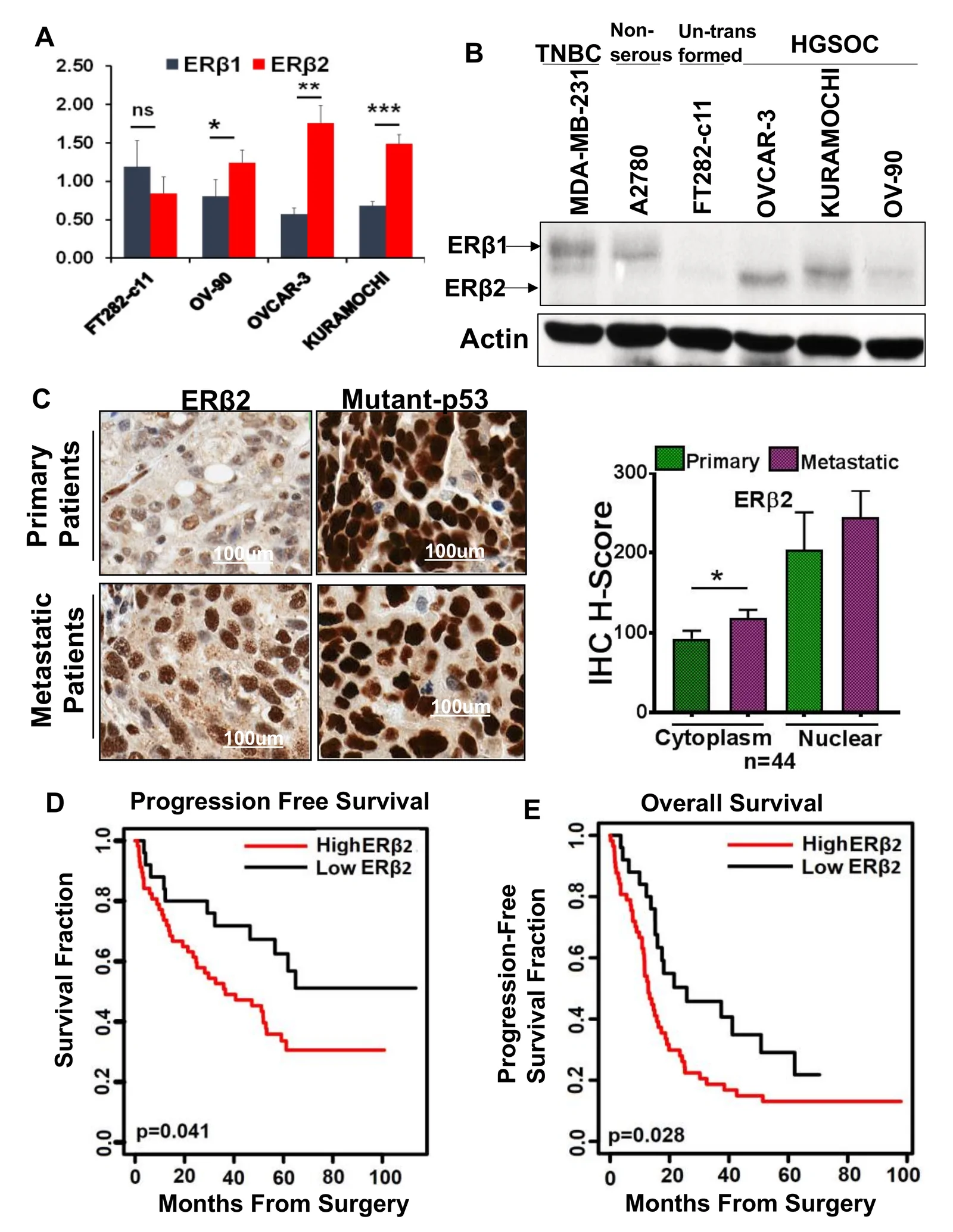
High ERβ2 expression is associated with tumor progression in HGSOC. (**A**) Total mRNA was isolated from FT282-c11 (untransformed fallopian tube cells), OVCAR3, KURAMOCHI, and OV-90 (HGSOC) using the Trizol method. The elative level of mRNA expressions was determined using quantitative real time PCR (qRT-PCR). Three independent experimental replicates were used for statistical analysis. The error bar represents SD and *p* values were analyzed using unpaired student *t* test. ns = non-significant, * = <0.005, ** = 0.001, and *** = <0.0001. (**B**) FT282-C11 (untransformed), OV90, OVCAR3, and KURAMOCHI (HGSOC cell lines), A2780 (non- serous ovarian cancer), and MDA-MB-231 (triple negative breast cancer /TNBC cells) were lysed in radio immunoprecipitation assay (RIPA) buffer. ERβ1 and ERβ2 protein expression was analyzed by immunoblotting with pan-ERβ antibody. (**C**) Left panel: ERβ2 and p53 Immunohistochemistry (IHC) staining of a typical tumor tissue core from a HGSOC patient tissue microarray (TMA). Right panel: Quantification of cytoplasmic and nuclear ERβ2 H-Score in primary versus peritoneal metastasis HGSOC patients. Statistical analysis was performed on H-sore from 44 patient primary tumors and their corresponding metastatic tumors. Error bars represent standard deviation (SD) and *p* values were determined using unpaired student *t* test. * = <0.005. (**D**,**E**) Kaplan–Meier survival curves in HGSOC patients showing tumors (*n* = 83) with high ERβ2 levels had worse prognosis in terms of both progression free survival (PFS) (**D**) and overall survival (OS) (**E**). Statistical analyses were performed in R4.0.3, all tests are two-sided and *p*-values less than 0.05 were considered significant. Survival analyses used Contal and O’Quigley’s method to select a cut point. The uncropped Western Blot images can be found in Figure S8.

**Figure 2:**
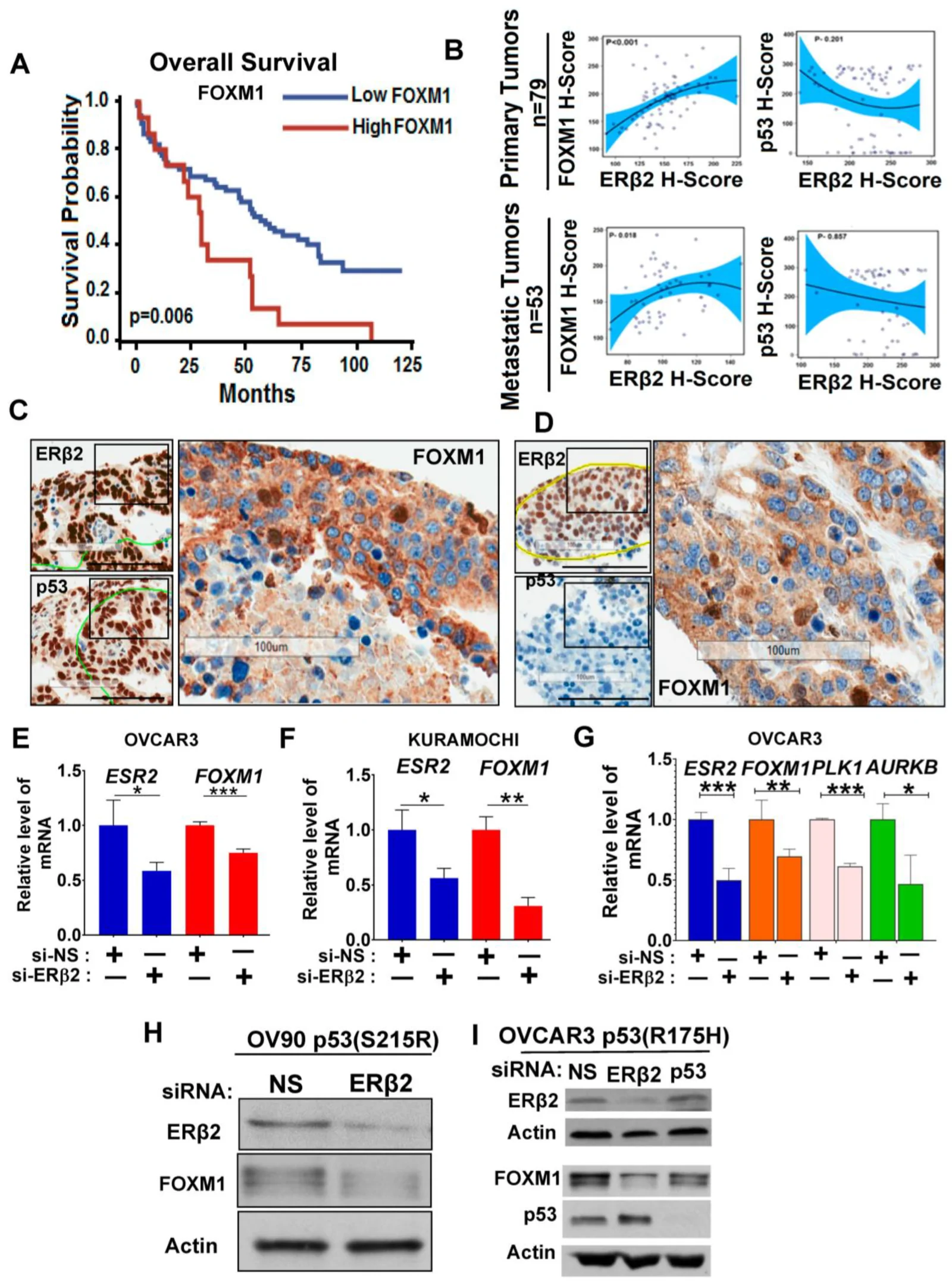
Upregulation of FOXM1 by ERβ2 is mutant p53-dependent. (**A**) Kaplan–Meier survival curves showing patients with HGSOC primary tumors expressing high FOXM1 (*n* = 79) had worse prognosis in terms of overall survival (OS). (**B**) Using the H-Scores, relationship between ERβ2 and FOXM1 or p53 in primary or metastatic tumors were analyzed using Spearman correlation methods, including 95% confidence bands for the correlation coefficient estimates. Correlation between ERβ2 & FOXM1 levels (left panel) and p53 &ER_2 levels (right panel) in primary (*n* = 79) (top panel) versus metastatic (*n* = 53) (bottom panel) in HGSOC tumors on TMA. The p values address the null hypothesis of no correlation between the two markers in each plot. Thep values have not been adjusted for multiple testing. (**C**,**D**) Representative IHC images of HGSOC patient tumors expressing (**C**) high and (**D**) low levels of ERβ2, p53 and FOXM1 (Scale bar: 100 mm). (**E**) OVCAR3 and (**F**) KURAMOCHI cells were treated with non-specific (si-NS) or ERβ2 specific siRNA (si-ERβ2) for 48 h, followed by analysis of ESR2 and FOXM1 transcripts by qRT-PCR. (**G**) OVCAR3 cells were transiently transfected with non-specific siRNA (si-NS) or ESR2-specific siRNA for 48 h. Post transfection, RNA levels of ESR2, FOXM1, and downstream targets of FOXM1(PLK1 and AURKB genes) were determined by qRT-PCR. Statistical analysis in (**E**–**G)**: Three independent experimental replicates were used for statistical analysis. The error bar represents SD, and p values were analyzed using an unpaired Student’s t-test. * = <0.005, ** = 0.001, and *** = <0.0001. (**H**) OV90 cells were transiently transfected with non-specific siRNA (si-NS) or ESR2-specific siRNA for 48 h. Expression of ERβ2, FOXM1 and actin proteins were analyzed by immunoblotting. (**I**) OVCAR3 cells were transiently transfected with non-specific siRNA (si-NS) or ESR2-specific siRNA for 48 h. Post transfection, expression of ERβ2, FOXM1, and p53 proteins were analyzed by immunoblotting. The uncropped Western blot images corresponding to (**H**,**I**) can be found in Figure S9.

For general comparison, triplicate data sets were analyzed by Student’s *t*-test and one-way ANOVA with multiple comparisons using GraphPad Prism 9 software (San Diego, CA, USA). Statistical analyses for generating Kaplan-Meier survival curves (Figure 1D,E) were performed in R4.0.3; all tests were two-sided and *p*-values less than 0.05 were considered significant. Survival analyses used Contal and O’Quigley’s method [40] to select a cut point. Based on the H-Scores from IHC, the relationship between ERβ2 and FOXM1 or p53 in primary or metastatic tumors were analyzed using Spearman correlation methods, including 95% confidence bands for the correlation coefficient estimates (Figure 2B). The *p* values address the null hypothesis of no correlation between the two markers in each plot. The *p* values on the plot are unadjusted.

## 3. Results

### 3.1. High ERβ2 Expression Is Associated with Tumor Progression in HGSOC

Isoforms of ERβ, such as ERβ1, ERβ2, and ERβ5 have been reported to be expressed in HGSOC with important roles in signaling pathways, cell cycle regulation and apoptosis [28,30,41,42,43].

To assess the relative expression levels of ERβ1 and ERβ2 in HGSOC, we determined their RNA levels in extracts of HGSOC cell lines OV90, OVCAR-3, and KURAMOCHI as compared to the expression in FT282-c11, an untransformed fallopian tube epithelial cell line [44]. Levels of the ERβ2 mRNA (as determined by quantitative real time PCR/qRT-PCR) was higher than ERβ1 mRNA levels in the HGSOC cells, but there was no significant difference in the isoform mRNA expression in FT282-c11 cells (Figure 1A). Similarly, ERβ2 protein levels were higher than ERβ1 levels in the HGSOC cell as compared to the levels in the untransformed FT282-c11 cells. Of note, in the non-serous ovarian cancer cell line A2780, ERβ1 levels were higher (Figure 1B). A triple negative breast cancer (TNBC) cell line, MDA-MB-231, where ERβ1 levels were higher than that of the ERβ2 levels is included as a positive control. ERβ1 and ERβ2 protein expression pattern in OVCAR3 and FT282-11 cells was further validated by transient expression of exogenous FLAG-ERβ1 and FLAG-ERβ2 and transient transfection of ERβ2 siRNA (Figure S3A–C). The specificity of the ERβ2 antibody was ascertained by knocking down ERβ2 with ERβ2-specific siRNA (Figure S3D). Consistent with these data, the IHC analysis of HGSOC tumor specimens on TMAs showed both the nuclear and cytoplasmic ERβ2 and p53 expression (Figure 1C). Furthermore, cytoplasmic expression of ERβ2 was higher in metastatic tumors (*n* = 44) as compared to primary tumors from which they progressed. However, p53 expression was similar in both the primary and metastatic tumors (Figure S2). We used IHC to determine the p53 status of tumors, as immunohistochemical analysis has been shown to be a robust method of inferring the presence of a p53 mutation in ovarian carcinomas [45]. A Kaplan–Meier analysis showed that high ERβ2 expression in primary tumors (*n* = 83) correlated with shorter PFS (Figure 1D) and OS (Figure 1E). ERα expression was very low both when analyzed by immunoblotting (Figure S1A) and by IHC in HGSOC tumors on the TMA (Figure S1B).

### 3.2. Upregulation of FOXM1 by ERβ2 Is Mutant p53-Dependent

Ovarian TCGA data have shown that the FOXM1 signaling is increased in HGSOC likely due to the universal presence of mutant p53 [2]. Therefore, we analyzed FOXM1 expression in our HGSOC patient tumor tissues by IHC on the TMAs. Kaplan Meier analysis showed that HGSOC patients with tumors (*n* = 83) with high FOXM1 levels had worse prognosis in terms of OS (*p* = 0.006) (Figure 2A).

Moreover, statistical analysis of IHC H-Score of ERβ2, FOXM1 and mutant p53 in HGSOC patient TMAs using Spearman correlation methods, including 95% confidence bands for the correlation coefficient estimates, showed that the cytoplasmic ERβ2 and FOXM1 protein had significant linear co-relation in primary (*p* < 0.001) as well as metastatic (*p* < 0.018) tumors (Figure 2B, upper and bottom left panels, respectively). However, no such correlation between the levels of nuclear ERβ2 and p53 was observed (Figure 2B, right panels). FOXM1 protein was highly expressed in the cytoplasm in the primary as well as in the metastatic tumors (Figure 2C and Figure S2). Additionally, ERβ2, mutant p53 and FOXM1 IHC staining data showed that high ERβ2 and high mutant p53 H-Score is consistent with high cytoplasmic FOXM1 expression (Figure 2C), whereas low ERβ2 and p53 expression led to reduced cytoplasmic expression of FOXM1 (Figure 2D). Based on these data, along with the reported ability of ERβ2 to drive antiapoptotic pathways in HGSOC [30], we explored if ERβ2 alone or in association with mutant p53 had any role in the regulation of FOXM1 in HGSOC. *FOXM1* mRNA levels were significantly reduced when ERβ2 was knocked down in OVCAR3 and KURAMOCHI cell lines (Figure 2E,F) suggesting a role for ERβ2 in the transcriptional regulation of FOXM1. Mutant p53 appeared to be necessary for enabling ERβ2 to upregulate downstream targets (PLK1 and AURKB) of FOXM1 as these genes were upregulated in OVCAR-3 cells harboring mutant p53 (Figure 2G). Consistent with the transcription data, depletion of ERβ2 led to a decrease in FOXM1 protein levels in OVCAR3 and OV90 cells expressing mutant p53 (Figure 2H). Furthermore, FOXM1 expression in OVCAR3 cells was decreased by depleting endogenous ERβ2 as well as mutantp53 (Figure 2I). These data suggest ERβ2 is necessary, but not sufficient, for activating *FOXM1* transcription. The co-dependency of ERβ2 and mutant p53 in regulating FOXM1 is further substantiated by the finding that combined depletion of ERβ2 and mutant p53 resulted in more reduction in FOXM1 protein levels than when ERβ2 alone was depleted (Figure S5A). Of note, neither DPN (an agonist of ERβ) nor Tamoxifen and PHTTP (antagonists of ERβ) affected FOXM1 levels in OVCAR3 cells (Figure S5B).

### 3.3. ERβ2 and Mutant p53 Physically Interact and Are Recruited to the FOXM1 Gene Promoter Leading to Activation of Transcription

**Figure 3:**
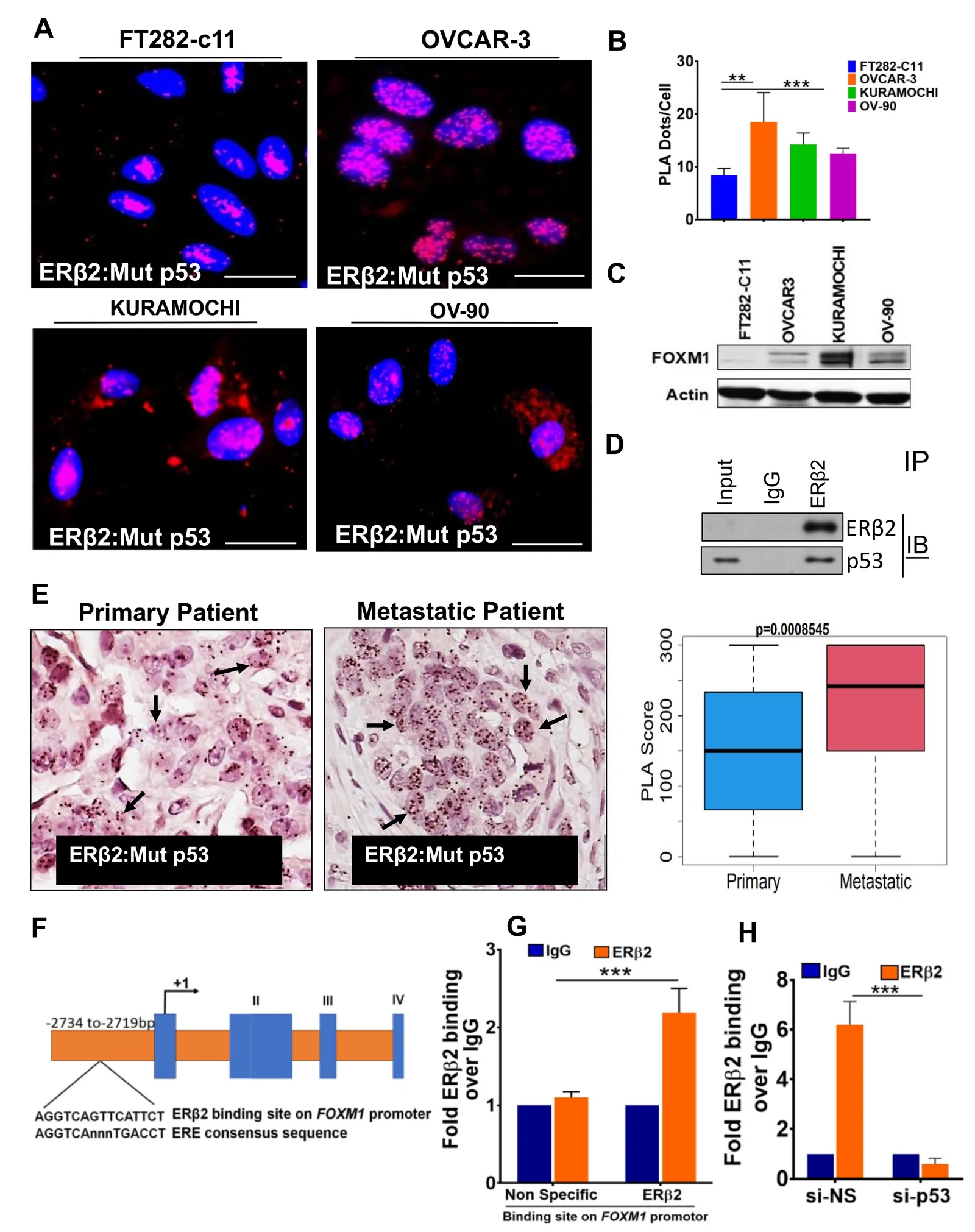
ERβ2 and mutant p53 physically interact and are recruited to the *FOXM1* gene promoter leading to activation of transcription. (**A**) ERβ2-p53 interaction was analyzed by proximity ligation assay (PLA) in FT282-c11, OVCAR3, KURAMOCHI, and OV90 cells. Scale bar = 20 µm. (**B**) Quantification of PLA dots by ImageJ software. Statistical analysis was performed on average number of dots (per 300 nuclei) in three independent experiments. Error bar represents SD and *p* values were analyzed using ANOVA test. ** = 0.001, and *** = <0.0001. (**C**) Immunoblot showing expression of endogenous FOXM1 in HGSOC cells. (**D**) Co-immunoprecipitation (Co-IP) of endogenous ERβ2 and mutant p53 was performed in OVCAR3 cells followed by immunoblotting with p53 and ERβ2 antibodies. (**E**) ERβ2–mutant p53 interaction in primary versus peritoneal metastatic HGSOC patient tumors on TMAs were assayed by Bright-field PLA. Representative images are shown. Quantification (by *t*-test) of PLA scores for ERβ2–mutant p53 interaction in primary versus peritoneal metastatic HGSOC patient tumors are shown on the right. PLA dots in 300 nuclei per patient tissue core in the TMA were manually counted using Bright-field microscopy. PLA scores >100 represent high interaction and dots <100 represent low interaction. (**F**) Schematic diagram of *FOXM1* gene promotor with ERβ2-binding site. (**G**) Quantitative chromatin immunoprecipitation (qChIP) with ERβ2-specific antibody or IgG (negative control) on *FOXM1* promoter was performed in OVCAR3 cells with or without depletion of ERβ2 for 48 h. (**H**) OVCAR3 cells transiently transfected with non-specific siRNA (si-NS) or TP53-specific siRNA. 48 h post-transfection, qChIP for ERβ2 or IgG (negative control) on *FOXM1* promoter was performed. Statistical analysis for data in Figure 3G, H was performed based on three independent experiments. Error bar represents SD and *p* values were analyzed using an unpaired Student’s *t*-test. *** = <0.0001. The uncropped Western Blot images can be found in Figure S10.

We had previously reported that ERβ binds directly to p53 in breast cancer cells and tissues [35]. Here we analyzed the physical interaction between ERβ2 and mutant p53 in HGSOC. A proximity ligation assay (PLA) demonstrated a robust interaction between ERβ2 and p53 *in situ* in OVCAR3, KURAMOCHI, and OV90 cells, and relatively lower interaction in the FT282-C11 cells (Figure 3A,B). The specificity of interaction was ascertained by knocking down ERβ2 in OVCAR3 and OV-90 cells followed by PLA (Figure S3E). Consistent with the interaction, FOXM1 levels were higher in OVCAR3, KURAMOCHI and OV90 cells compared to that in FT282-c11 cells (Figure 3C). The physical interaction between ERβ2 and mutant p53 in OVCAR3 cells was orthogonally confirmed by co-immunoprecipitation assay (Figure 3D). Furthermore, we investigated if ERβ2-mutant p53 interaction occurs in HGSOC patient tumors, and if so, whether the extent of interaction differs between primary versus metastatic tumors. Toward this goal, we performed PLA on TMAs containing both primary (*n* = 88) and metastatic (*n* = 56) tumor tissues arrayed in triplicate. Importantly, metastatic tumors had more intense PLA signals compared to primary tumors (Figure 3E). The difference was highly significant as shown in the quantitation plot on the right. Importantly, p53 levels were high (consistent with mutant p53) along with elevated PLA signals (indicating ERβ2-mutant p53 interaction) in HGSOC (Figure S4A), while the p53 levels were low (consistent with wild type p53) and correspondingly negative for PLA signals (indicating lack of interaction between ERβ2 and wild type p53) in LGSOC (Figure S4B). Next, for testing the hypothesis that ERβ2 and mutant p53 bind to the *FOXM1* promoter leading to the upregulation of *FOXM1* transcription, we performed a quantitative chromatin immunoprecipitation (q-ChIP) assay. We used the JASPAR database [46] to identify a consensus ER response element (ERE) (−2734 to −2719) in the *FOXM1* gene promoter (Figure 3F). ERβ2 bound to this site, but not to a non-specific region in the promoter (Figure 3G). Moreover, the binding was abolished when mutant p53 was knocked down (Figure 3H). These data on the interaction of ERβ2-mutant p53 complex on *FOXM1* promoter chromatin are consistent with the co-dependency of ERβ2 and mutant p53 in upregulating FOXM1 protein levels noted before (Figure 2I).

### 3.4. ERβ2 Activates FOXM1 Expression and Confers Resistance to Carboplatin

**Figure 4:**
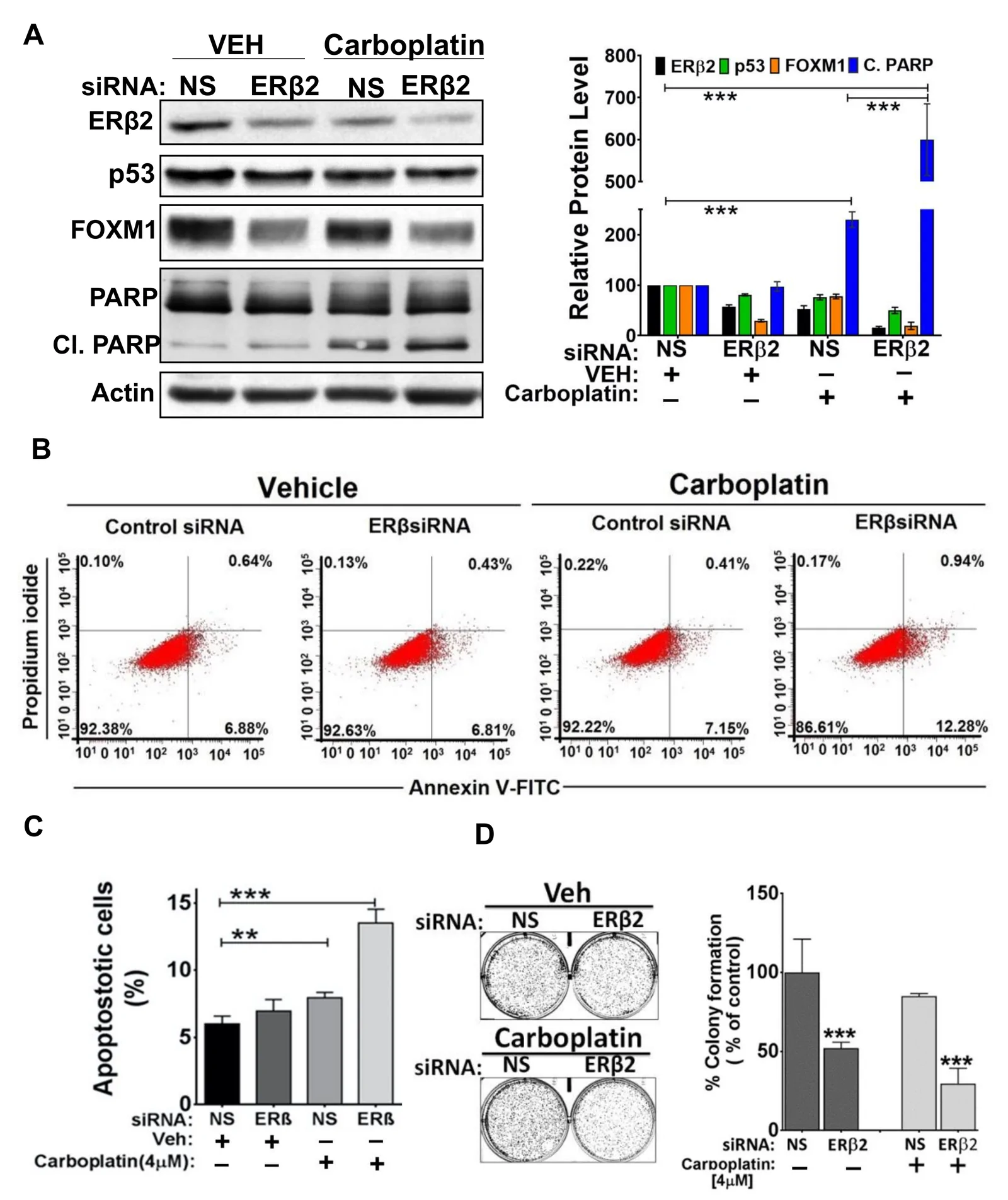
ERβ2 activates FOXM1 expression and confers resistance to carboplatin treatment. (**A**) OVCAR3 cells were transiently transfected with non-specific (ns-siRNA) or ERβ2-specific siRNA for 48 h, followed by carboplatin (4 µM) treatment for 24 h. Post treatment, cells were harvested and expression levels of FOXM1, p53, ERβ2, PARP and cleaved PARP were determined by immunoblotting. A representative immunoblot from three experimental replicates is shown. Right panel shows the quantification of FOXM1, p53, ERβ2, PARP and cleaved PARP after normalization to actin levels. Statistical analysis was performed on average quantity of each protein on the immunoblot from three independent experiments. Error bar represents SD and *p* values were analyzed using ANOVA test. *** = <0.0001. (**B**) Flow cytometry analysis of OVCAR3 cells post-transfection with control siRNA or ERβ2 siRNA for 48 h and treated with vehicle or carboplatin (4 µM). Cells were double stained with Annexin V-FITC and Propidium Iodide (PI) for apoptosis assay. (**C**) Quantitation of data shown in B. Bar graph shows fold change of Annexin +/PI—cells normalized with untreated control. Statistical analysis was performed using average quantity of annexin V from three independent experiments. Error bar represents SD and *p* values were analyzed using ANOVA test. ** = 0.001, and *** = <0.0001. (**D**) OVCAR3 cells 48 h post-transfection with non-specific (ns-siRNA) or ERβ2-specific siRNA were treated with carboplatin (4 µM). Treated cells were re-seeded, and after nine days colonies were stained with crystal violet and quantified using a Bright-field microscope. Right panel shows the quantification colonies of OVCAR3 cells. Statistical analysis was performed using average percentage of colony formation from three independent experiments. Error bar represents SD and *p* values were analyzed using an unpaired Student’s *t*-test. *** = <0.0001 Error bars represent standard deviation (SD). Veh: Vehicle. The uncropped Western Blot images can be found in Figure S11.

FOXM1 has been reported to confer resistance to chemotherapeutic agents such as platinum drugs in epithelial ovarian cancer [47]. We hypothesized that FOXM1 expression could be upregulated by ERβ2, leading to the resistance to carboplatin, an agent frequently used to treat HGSOC. To test this possibility, OVCAR3 cells were treated with multiple doses of carboplatin without and with siRNA-mediated transient depletion of ERβ2 in OVCAR3 cells (Figure S6A,B). Immunoblotting data showed that treatment with carboplatin (4 µM) in ERβ2-depleted OVCAR3 cells decreased FOXM1 protein expression and increased carboplatin sensitivity resulting in up-regulated apoptosis as indicated by a sixfold increase in the cleaved PARP protein level (Figure 4A). Similar data were also observed on carboplatin and cisplatin treatment with the combination of ERβ2 knockdown in OV90 cells (Figure S6C). Consistent with these data, an Annexin V assay showed that ERβ2 depletion along with carboplatin treatment increased apoptosis more than two-fold compared to vehicle treated cells (Figure 4B; quantitation in Figure 4C). Furthermore, a cell survival assay demonstrated that treatment with 4 μM carboplatin 72 h post-transfection with ERβ2 siRNA resulted in decreased cell survival as compared to cells treated with vehicle (Figure 4D).

### 3.5. Disruption of ERβ2-Mutant p53 Crosstalk Leads to HGSOC Cells Apoptosis

**Figure 5:**
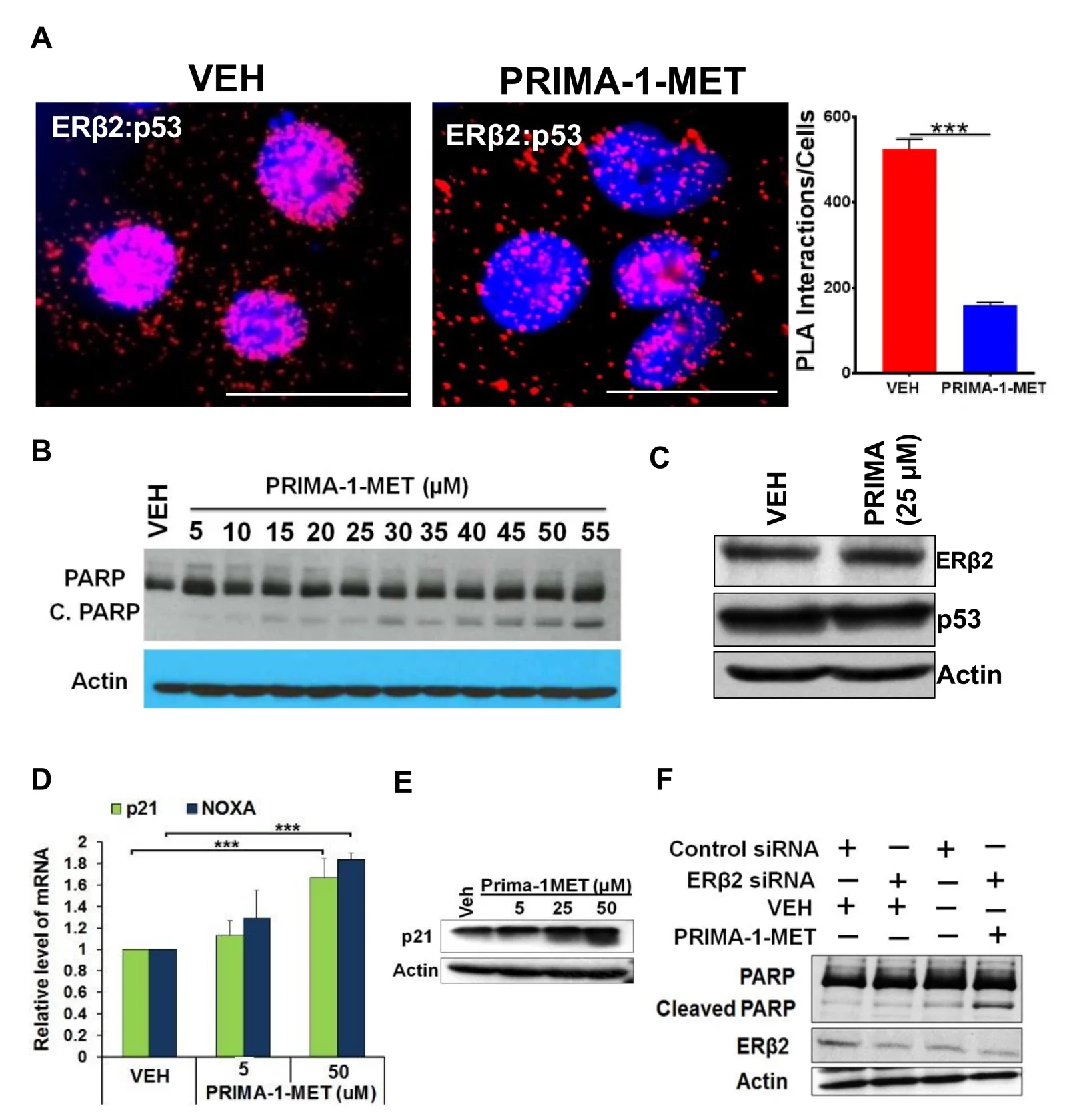
Disruption of ERβ2-mutant p53 interaction leads to apoptosis. (**A**) OVCAR3 cells treated with vehicle (left panel) or 25 µM PRIMA-1MET. Post-24 h treatment, PLA was performed for ERβ2-mutant p53 interaction (Scale bar = 20 µm). Right panel: Quantification of the ERβ2: p53 PLA dots with ImageJ software. Statistical analysis was performed on average number of dots (per 300 nuclei) in three independent experiments. Error bar represents SD and p-values were analyzed using ANOVA test. *** = <0.0001. (**B**) Dose titration of PRIMA-1MET in OVCAR3 cells and determining apoptosis by PARP cleavage assay using immunoblotting. (**C**) Expression of ERβ2 and p53 proteins in OVCAR3 cells 24 h post-treatment with PRIMA-1MET (25 µM) was analyzed by immunoblotting. (**D**) OVCAR3 cells were treated with vehicle or different doses of PRIMA-1MET (5 µM, 50 µM) for 24 h and transcripts of p21 and NOXA were analyzed with qRT-PCR. Statistical analysis was performed on three independent experiments. Error bar represents SD and p-values were analyzed using ANOVA test *** = <0.0001. (**E**) OVCAR3 cells were treated with vehicle or different doses (5 µM, 25 µM, 50 µM) of PRIMA-1MET for 24 h followed by determination of p21 protein expression by immunoblotting. (**F**) OVCAR3 cells were transfected with or without ERβ2 siRNA, followed by treatment with vehicle or 25 µM PRIMA-1MET for 24 h. Post treatment, PARP, Cleaved PARP and ERβ2 expression was analyzed by immunoblotting. VEH: Vehicle. The uncropped Western Blot images can be found in Figures S12 and S13.

**Figure 6:**
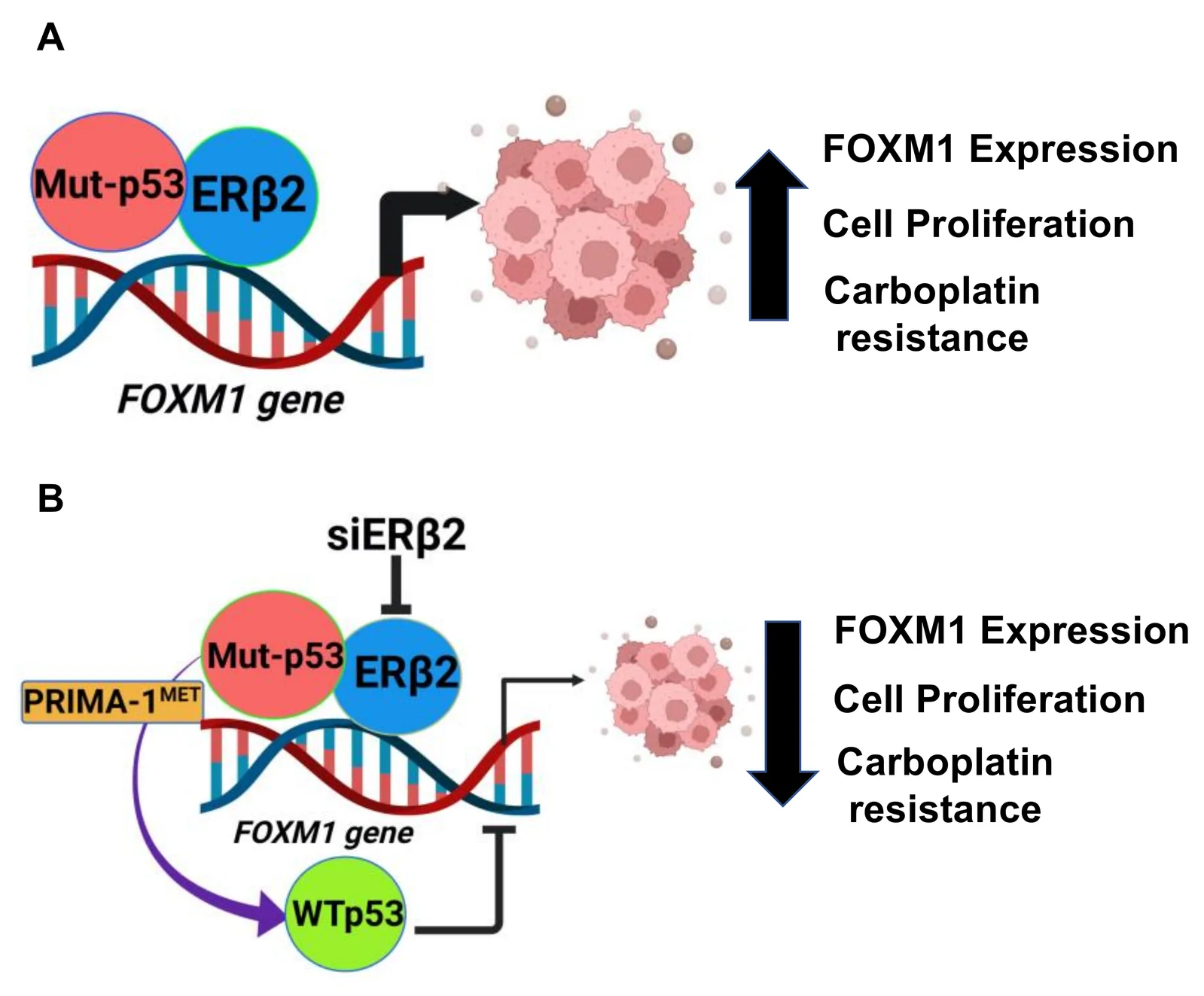
Working model for ERβ2-p53-FOXM1 signaling axis and therapeutic resistance in HGSOC. (**A**) Working model where ERβ2 interacting with mutant p53 upregulates *FOXM1* transcription leading to increased proliferation and therapeutic resistance. (**B**) Model for reversal of effects in A when *FOXM1* transcription gets downregulated by the combined action of ESR2-specific siRNA and PRIMA-1^MET^. The figure was created with “Biorender.com (access on: 13 November 2021).

Based on our findings that ERβ2-mutant p53 interaction leads to increased proliferation and resistance to chemotherapy, we hypothesized that disruption of this interaction would decrease the pro-proliferation effects in HGSOC cells. PRIMA-1 (p53 reactivation and induction of massive apoptosis) and its methylated form PRIMA-1^MET^ are small molecules capable of converting mutant p53 to active wild type conformation and have been shown to overcome drug resistance in ovarian cancer cells [48,49]. First, we tested the effect of PRIMA-1^MET^ on the interaction between ERβ2 and mutant p53 in OVCAR3 cells. The interaction was decreased considerably in response to the treatment (Figure 5A). This disruption of interaction by the drug leads to increased apoptosis (as evidenced by PARP cleavage) in a dose-dependent manner in OVCAR3 (Figure 5B) and OV90 cells (Figure S6). Importantly, neither the levels of ERβ2 nor mutant p53 were changed in response to the PRIMA-1^MET^ treatment (Figure 5C), whereas both the RNA (Figure 5D) and protein (Figure 5E) levels of the prototypic p53-target and anti-proliferation protein p21 (CDKN1A) were increased in response to the drug treatment. Transcript levels of NOXA, a pro-apoptotic gene, was also increased by PRIMA-1^MET^ (Figure 5D). Furthermore, the combination of ERβ2 depletion with PRIMA-1^MET^ treatment led to a further increase in apoptosis as indicated by increased PARP cleavage in OVCAR3 cells (Figure 5F). A similar increase in apoptosis was also observed in OV90 cells (Figure S7) Based on our data, a potential model for the mechanism underlying drug resistance in HGSOC is shown in Figure 6A. Figure 6B shows how depleting pro-tumorigenic ERβ2 in combination with PRIMA-1^MET^ that converts mutant p53 to WT conformation elicits increased apoptosis and overcomes resistance to carboplatin.

## 4. Discussion

To the best of our knowledge, this is the first report on the crosstalk between ERβ2 and mutant p53 to upregulate *FOXM1* expression, proliferation, and therapeutic resistance in HGSOC cells and patient tumor tissues. *FOXM1* was previously reported to be a target of transcriptional repression by wild type p53 [32,33] and the repression by p53 was shown to be E2F-dependent [50]. Analysis of the TCGA of HGSOC showed that there is almost universal (~96%) expression of mutant or null alleles of p53 in these tumors along with high levels of *FOXM1* and the activation of pro-proliferation pathways downstream [2]. In addition to being regulated by p53, *FOXM1* has been reported to be regulated by ERβ as well. However, results from these studies have been inconclusive. While *FOXM1* was one of the genes downregulated when ERβ1 was exogenously expressed in breast cancer cells [51], it was not affected by ERβ1 overexpression in another study [52]. Neither study looked at the effect of ERβ isomers such as ERβ2 that has been suggested to be pro-oncogenic. ERβ2 localized to the mitochondria was reported to interact with BAD and inhibit apoptosis [30]. It is becoming increasingly clear that understanding the role of wild type and mutant p53 is of high relevance to the etiology and treatment of HGSOC [13,14]. Targeting mutant p53 interaction with partner proteins is a promising therapeutic strategy [53]. ERβ’s oncogenic role may not only be isoform specific but may also depend on the mutational context of partner proteins such as p53 [35]. Here we demonstrate that ERβ2, but not ERβ1, is the prominent ERβ form in HGSOC cells, whereas ERβ1 is the major isoform expressed in the non-serous cell line A2780. The normal fallopian tube cell line FT282-c11 did not express much of either isoform. Furthermore, ERβ2 levels were higher in metastatic HGSOC compared to the primary tumors they had evolved from and were correlated with worse PFS and OS. Of note, ERα levels were relatively low in HGSOC patient tissues we used for our studies, consistent with the report of a large study conducted by the Ovarian Tumor Tissue Analysis consortium in 1742 HGSOC cases that showed that ERα was not associated with improved HGSOC survival [18]. Importantly, there was positive correlation between ERβ2 expression and *FOXM1* levels in HGSOC patient tumors, and higher levels of *FOXM1* correlated with low OS. Our molecular analyses revealed a co-dependency between ERβ2 and mutant p53 in upregulating FOXM1 in HGSOC cells. In situ PLA showed that ERβ2 physically interacted with mutant p53 both in the HGSOC cells and in patient tumors. The importance of such co-dependency and interaction in upregulating *FOXM1* transcription was ascertained by a qChIP assay that showed mutant p53-depencent ERβ2 binding to the *FOXM1* gene promoter. FOXM1 drives ovarian oncogenesis in multiple ways including inducing resistance to chemotherapeutic agents such as carboplatin [34]. Our data that the ablation of ERβ2 increases sensitivity of OVCAR3 cells to carboplatin by increasing apoptosis and decreasing cell proliferation suggest that ERβ2 in concert with mutant p53 is a major driver of carboplatin resistance in HGSOC. This conclusion is further strengthened by the observation that conversion of mutant p53 to wild type conformation by PRIMA-1^MET^ resulted in decreased ERβ2-p53 interaction leading to a dose-dependent increase in p21 (*CDKNIA*) and *NOXA* expression and increased apoptosis. Moreover, when PRIMA-1^MET^ treatment was combined with ERβ2 knockdown, there was further enhancement of apoptosis. These observations led to a working model where ERβ2 interacting with mutant p53 upregulates *FOXM1* transcription leading to increased proliferation and therapeutic resistance (Figure 6A). This effect is reversed when *FOXM1* transcription gets downregulated by the combined action of ERβ2-specific siRNA and PRIMA-1^MET^ (Figure 6B).

Although the importance of ERβ in ovarian cancer has been widely recognized, the issue of whether it is pro- or anti-oncogenic remains unsettled [15,54]. The lack of well-defined molecular studies to determine the major oncogenic ERβ isoform that is predominantly expressed in HGSOC has been mainly responsible for disparate reports on the role of ERβ. The current study at least partially resolves this controversy. Furthermore, our study shines light on the importance of proteins such as ERβ that partner with mutant p53 either to drive or suppress oncogenesis depending on a particular cancer context. In the case of HGSOC, ERβ2 cooperates with mutant p53 to drive oncogenesis and drug resistance. Of note, although HGSOC and TNBC share several molecular similarities, our data showed that unlike in HGSOC cells, levels of ERβ1 are higher than that of ERβ2 in the TNBC cell line MDA-MB-231. Further studies are needed to decipher whether this is limited to a few cell lines or is a general difference between HGSOC and TNBC cells, and if so, its differential roles in driving these two types of aggressive cancers. It is important to note that although ERβ2 interacted with the p53 mutants expressed in the cell lines and tumor tissues we used for this study, it is quite possible that ERβ2 may not interact with all p53 mutants, as different mutations of p53 have been reported to affect tumor growth and progression differently. Therefore, stratifying HGSOC based on heterogeneity of p53 mutations and ERβ could be promising in developing new therapeutic strategies.

## 5. Conclusions

This study reveals the interaction between ERβ2 and mutant p53 and functional co-dependency in HGSOC cell lines and patient tumors. We have identified a novel ERβ2-mutant p53-FOXM1 signaling axis that drives increased proliferation, inhibition of apoptosis, and resistance to carboplatin therapy in HGSOC cell lines and affects the DFS and OS of patients. Drug-induced conversion of mutant p53 to wild type conformation when combined with depletion of ERβ2 mitigates these effects. A working model based on these observations is shown in Figure 6. These findings have important clinical implications and could trigger new mechanistic studies on the role of ERβ and mutant p53 toward developing new therapeutic strategies against HGSOC. An in-depth understanding of the molecular mechanisms by which different p53 mutations and different isoforms of ERβ crosstalk and impinge on driving ovarian oncogenesis should further refine these strategies.

## Data Availability

Data sharing is not applicable to any data included in this article.

## Supplementary Materials

The following supporting information can be downloaded at: https://www.mdpi.com/article/10.3390/cancers14051120/s1, Figure S1: ERβ, but not ERα, is the major hormone receptor in HGSOC; Figure S2: Immunohistochemistry (IHC) H Scores of FOXM1 and p53 in primary versus metastatic patient tumors; Figure S3: Induction of apoptosis by carboplatin and cisplatin; Figure S4: Protein levels of ERβ2 and p53 (IHC) and interaction between ERβ2 and p53 in HGSOC (A) and LGSOC (B); Figure S5A: Both ERβ2 and mutant p53 are necessary to upregulate FOXM1; Figure S5B: Effect of ERβ ligands on FOXM1 expression; Figure S6: Induction of apoptosis by carboplatin and cisplatin; Figure S7: Treatment with PRIMA-1MET induces apoptosis in OV-90 cells; Figures S8, S9, S19, S11, S12 and S13: Uncropped Western blot images; Table S1: Oligonucleotide primers; Table S2: Antibodies.
