# Correction: Oturkar et al. Estrogen Receptor-Beta2 (ERβ2)–Mutant p53–FOXM1 Axis: A Novel Driver of Proliferation, Chemoresistance, and Disease Progression in High Grade Serous Ovarian Cancer (HGSOC). *Cancers* 2022, *14*, 1120

**DOI:** 10.3390/cancers18142360

**Published:** 2026-07-22

**Authors:** Chetan C. Oturkar, Nishant Gandhi, Pramod Rao, Kevin H. Eng, Austin Miller, Prashant K. Singh, Emese Zsiros, Kunle O. Odunsi, Gokul M. Das

**Affiliations:** 1 Department of Pharmacology and Therapeutics, Roswell Park Comprehensive Cancer Center, Buffalo, NY 14263, USA; chetan.oturkar@roswellpark.org (C.C.O.); ngandhi@carisls.com (N.G.); pramodra@buffalo.edu (P.R.); 2 Department of Biostatistics & Bioinformatics, Roswell Park Comprehensive Cancer Center, Buffalo, NY 14263, USA; kevin.eng@roswellpark.org (K.H.E.); austin.miller@roswellpark.org (A.M.); 3 Genomic Shared Resource, Roswell Park Comprehensive Cancer Center, Buffalo, NY 14263, USA; prashant.singh@roswellpark.org; 4 Department of Gynecologic Oncology, Roswell Park Comprehensive Cancer Center, Buffalo, NY 14263, USA; emese.zsiros@roswellpark.org (E.Z.); odunsia@bsd.uchicago.edu (K.O.O.)

**Update to Figure**

In Figure 2 [1], the information on ERβ2 and FOXM1 expression in Figure 2H for OVCAR3 cells overlapped with the data presented in Figure 2I. In addition, the ERβ2 panel in Figure 2H for OVCAR3 cells was inadvertently flipped vertically. This error was overlooked in the original figure legend. The corrected legend appears below following deletion of the left OVCAR3 panel in Figure 2H. The authors state that the scientific conclusions are unaffected. This correction was approved by the Academic Editor. The original publication has also been updated.

Corrected Figure 2:

## Figures and Tables

**Figure 2:**
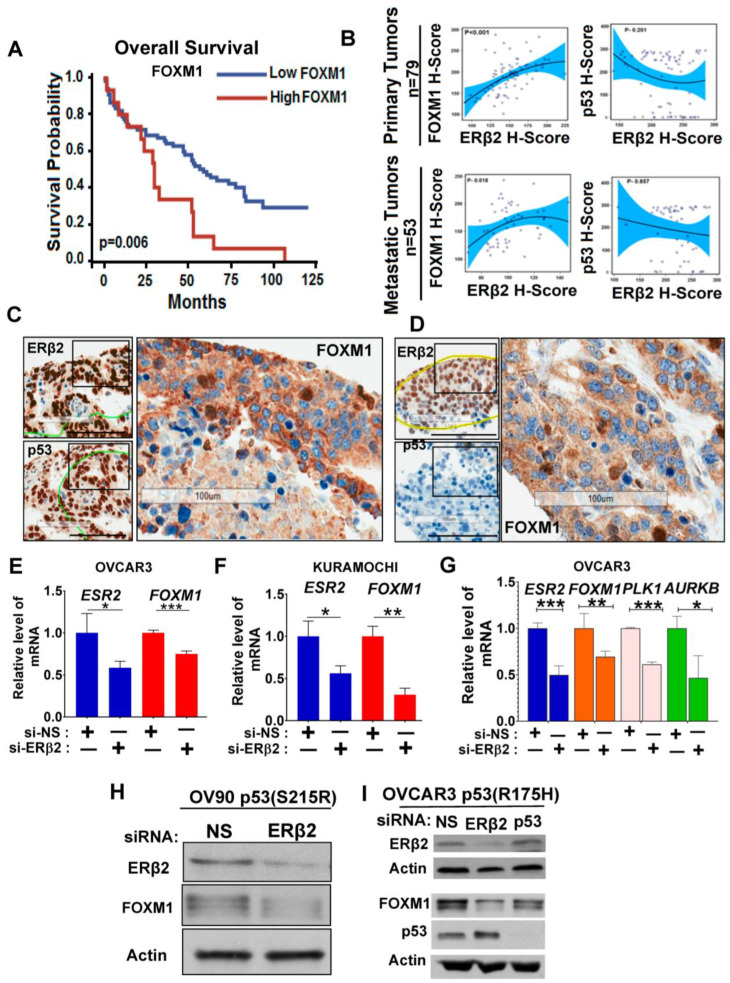
Upregulation of FOXM1 by ERβ2 is mutant p53-dependent. (**A**) Kaplan–Meier survival curves showing patients with HGSOC primary tumors expressing high FOXM1 (*n* = 79) had worse prognosis in terms of overall survival (OS). (**B**) Using the H-Scores, relationship between ERβ2 and FOXM1 or p53 in primary or metastatic tumors were analyzed using Spearman correlation methods, including 95% confidence bands for the correlation coefficient estimates. Correlation between ERβ2 & FOXM1 levels (left panel) and p53 &ER_2 levels (right panel) in primary (*n* = 79) (top panel) versus metastatic (*n* = 53) (bottom panel) in HGSOC tumors on TMA. The p values address the null hypothesis of no correlation between the two markers in each plot. Thep values have not been adjusted for multiple testing. (**C**,**D**) Representative IHC images of HGSOC patient tumors expressing (**C**) high and (**D**) low levels of ERβ2, p53 and FOXM1 (Scale bar: 100 mm). (**E**) OVCAR3 and (**F**) KURAMOCHI cells were treated with non-specific (si-NS) or ERβ2 specific siRNA (si-ERβ2) for 48 h, followed by analysis of ESR2 and FOXM1 transcripts by qRT-PCR. (**G**) OVCAR3 cells were transiently transfected with non-specific siRNA (si-NS) or ESR2-specific siRNA for 48 h. Post transfection, RNA levels of ESR2, FOXM1, and downstream targets of FOXM1(PLK1 and AURKB genes) were determined by qRT-PCR. Statistical analysis in (**E**–**G)**: Three independent experimental replicates were used for statistical analysis. The error bar represents SD, and p values were analyzed using an unpaired Student’s t-test. * = <0.005, ** = 0.001, and *** = <0.0001. (**H**) OV90 cells were transiently transfected with non-specific siRNA (si-NS) or ESR2-specific siRNA for 48 h. Expression of ERβ2, FOXM1 and actin proteins were analyzed by immunoblotting. (**I**) OVCAR3 cells were transiently transfected with non-specific siRNA (si-NS) or ESR2-specific siRNA for 48 h. Post transfection, expression of ERβ2, FOXM1, and p53 proteins were analyzed by immunoblotting. The uncropped Western blot images corresponding to (**H**,**I**) can be found in Figure S9.
