# Supplementary material for: Estrogen Receptor-Beta2 (ERβ2)–Mutant p53–FOXM1 Axis: A Novel Driver of Proliferation, Chemoresistance, and Disease Progression in High Grade Serous Ovarian Cancer (HGSOC)
Source: Cancers (Basel). 2022 Feb 22;14(5):1120. doi: 10.3390/cancers14051120 (PMC8909529; doi:10.3390/cancers14051120)
Supplement: Supplementary file 1 [file cancers-14-01120-s001.zip › cancers-1561651-supplementary.pdf]

cancers

MDPI

Supplementary Materials: Estrogen Receptor-beta2 (ERβ2)–Mutant p53–FOXM1 Axis: A Novel Driver of Proliferation, Chemoresistance, and Disease Progression in High Grade Serous Ovarian Cancer (HGSOC)

Chetan C. Oturkar, Nishant Gandhi, Pramod Rao, Kevin H. Eng, Austin Miller, Prashant K. Singh, Emese Zsiros, Kunle O. Odunsi and Gokul M. Das

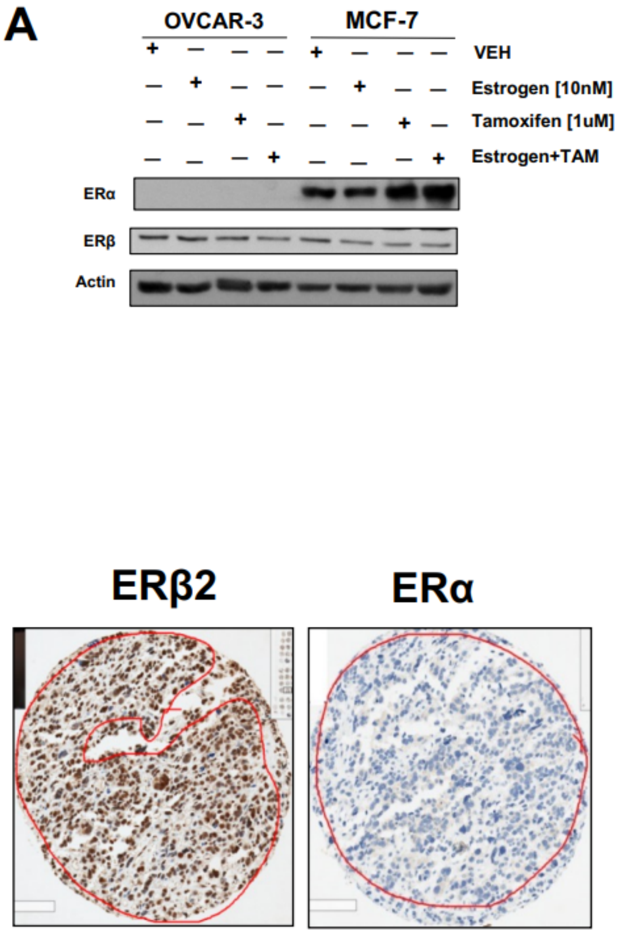

Figure S1. ERβ, but not ERα, is the major hormone receptor prevalent in HGSOC. (**A**) OVCAR-3 and MCF-7 cells were treated with vehicle, 17β-Estradiol (10 nM) and Tamoxifen (1 μM) for 24 hours followed by immunoblotting with ERα and ERβ antibodies. (**B**) Representative images of IHC staining for ERβ2 and ERα in HGSOC patient tumors on a TMA.

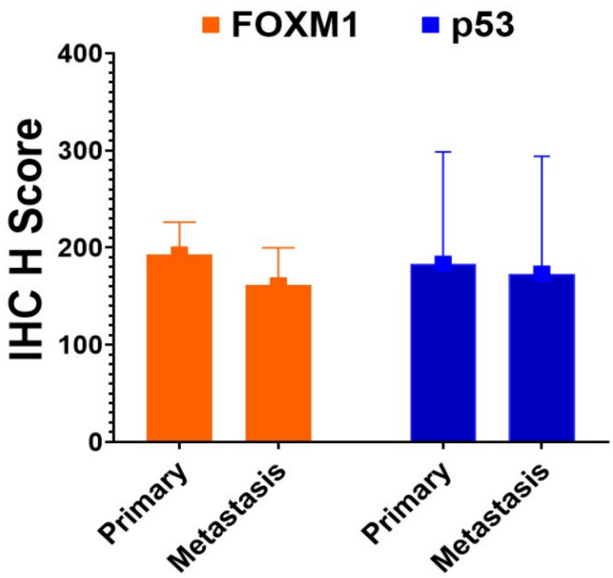

Figure S2. Comparison of Immunohistochemistry (IHC) H Scores of FOXM1 and p53 in primary versus metastatic patient tumors. IHC H-Score comparison of cytoplasmic FOXM1 and nuclear p53 in primary (n = 44) and corresponding metastatic tumors (n = 44) on HGSOC patient TMA.

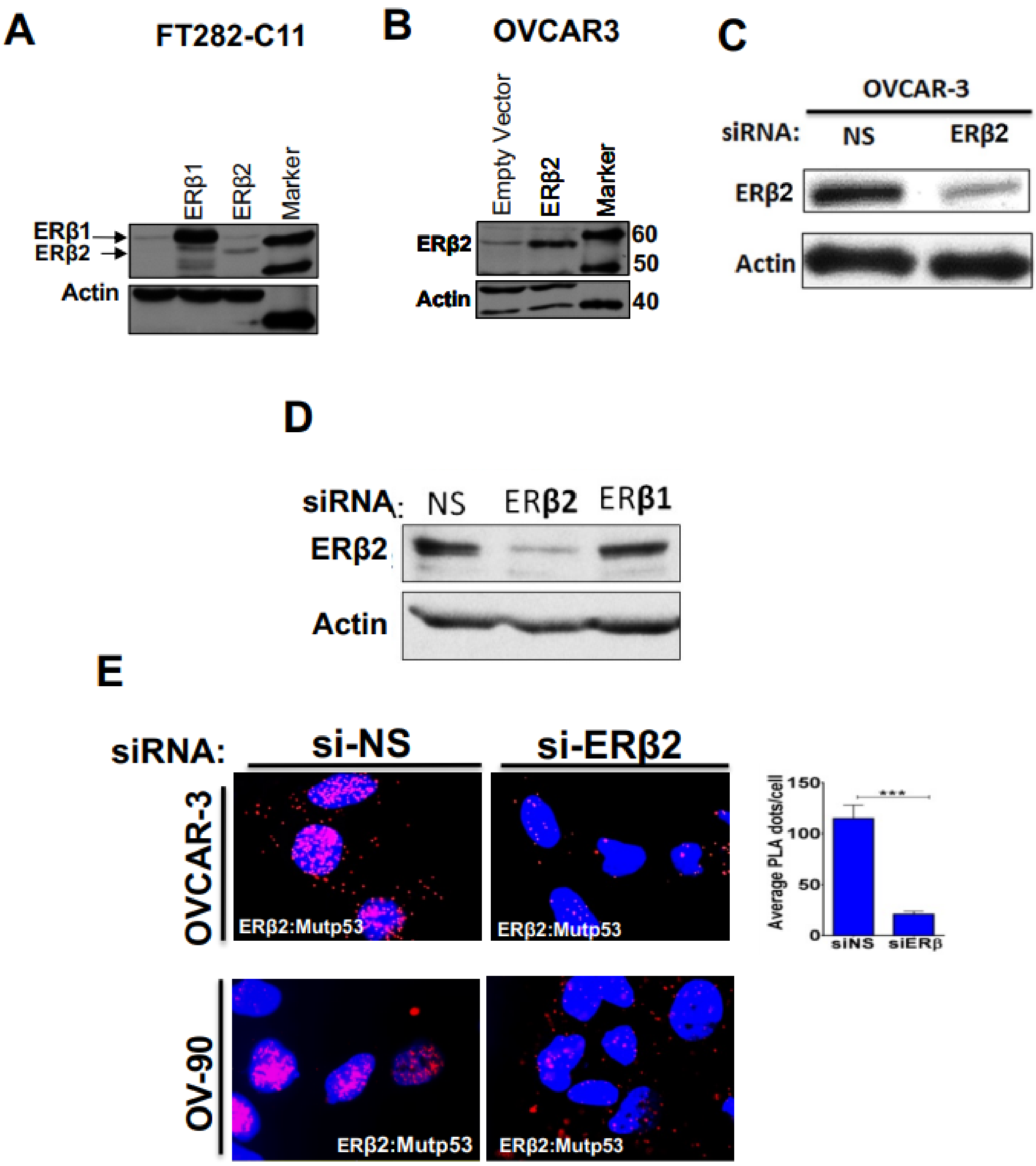

Figure S3. Ascertaining specificity of ERβ2 antibody and ERβ2-mutant p53 interaction. FT282-C11 (**A**) and OVCAR3 (**B**) were transfected with FLAG vector, FLAG-ERβ1 and FLAG-ERβ2 for 48 hours followed by immunoblotting with pan-ERβ antibody. (**C**) OVCAR-3 cells were transiently transfected with NS-siRNA and ERβ2-specific siRNA for 48 hours followed by immunoblotting with ERβ2 antibody. (**D**) OVCAR3 cells were transfected with non-specific siRNA (NS), ERβ1-specific or ERβ2-specific siRNAs and cell lysates were prepared 48 hours post-transfection followed immunoblotting with ERβ2 antibody. (**E**) Specificity of ERβ2-mutant p53 interaction in OVCAR3 and OV90 cells was ascertained by performing PLA without and with knocking down ERβ2. Statistical analysis was performed on average number of dots (per 300nuclei) in three independent experiments. Error bar represents SD and p values were analyzed using ANOVA test. *** = < 0.0001.

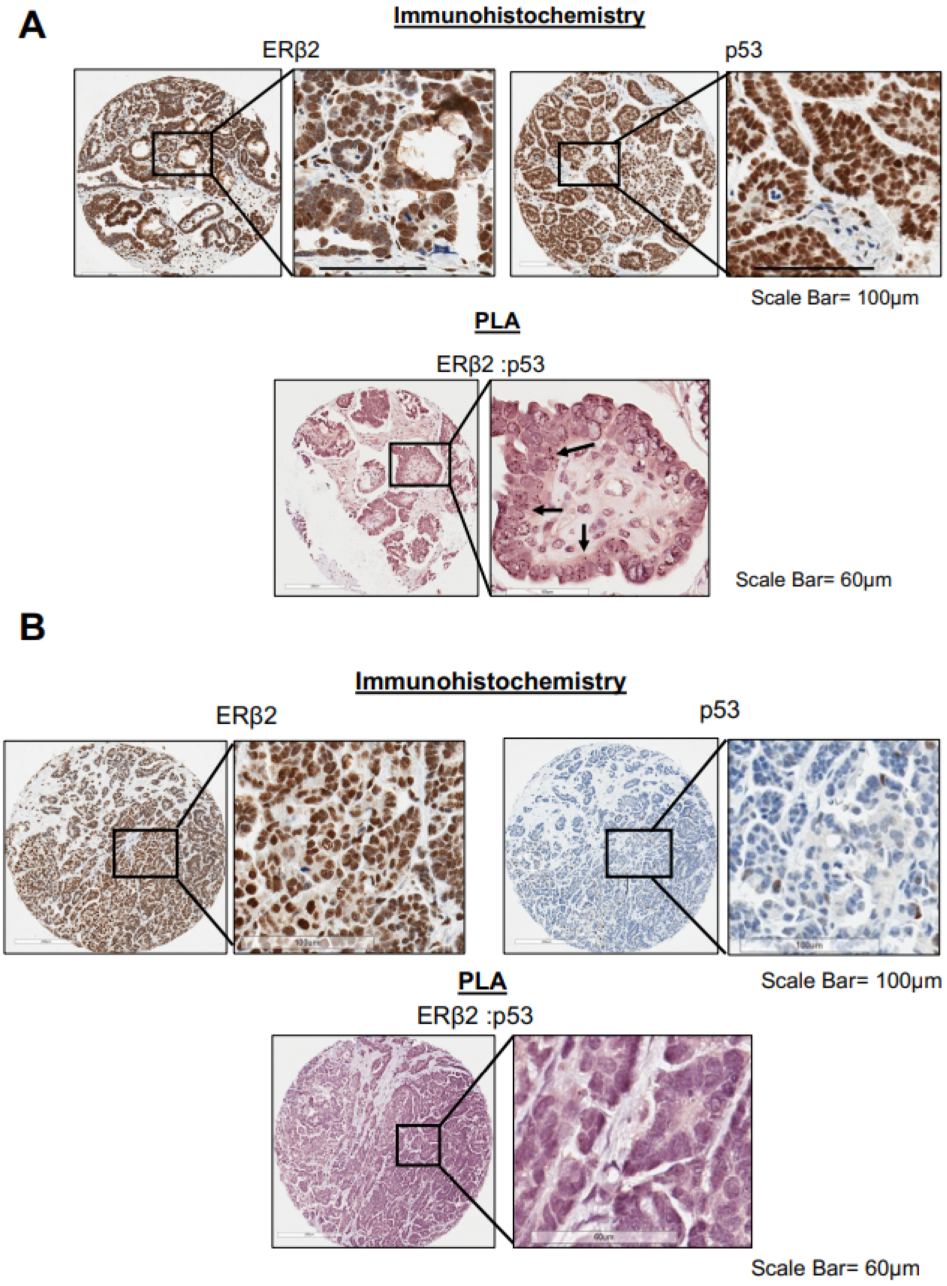

Figure S4. Protein levels of ERβ2 and p53 (IHC) and interaction between ERβ2 and p53 in HGSOC (**A**) and LGSOC (**B**). A. Representative images of IHC staining for ERβ2 and p53 and proximity ligation assay (for ERβ2-p53 interaction in HGSOC patient tumor tissue. Dots representing PLA signals are pointed by arrow. Scale Bar = 100 µM for IHC and 60 µM for PLA. B. Representative images of IHC staining for ERβ2 and p53 and proximity ligation assay for ERβ2-p53 interaction in LGSOC patient tumor tissue. Dots representing PLA signals are pointed by arrow. Scale Bar = 100 µM for IHC and 60 µM for PLA.

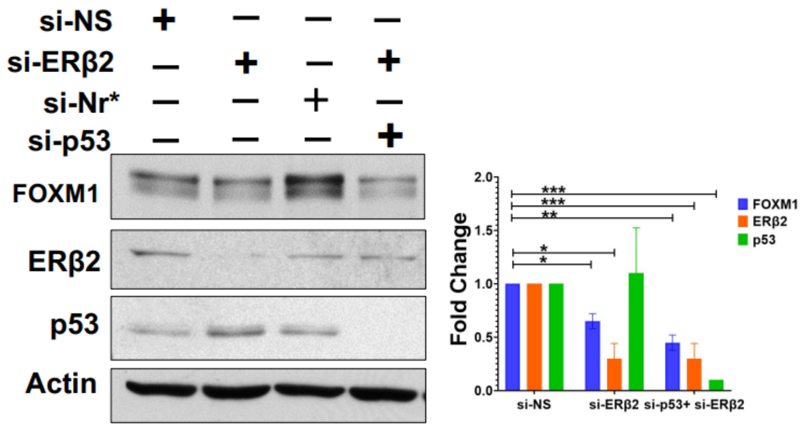

Figure S5. A. Both ERβ2 and mutant p53 are necessary to upregulate FOXM1. OVCAR-3 cells were transfected with either control non-specific siRNA (si-NS), si-ERβ2, si-un-related (si-Ur*), and si-p53 for 48 hours. Post transfection expression of ERβ2, p53, FOXM1 proteins was analyzed by immunoblotting. Quantification graph is shown on the right.

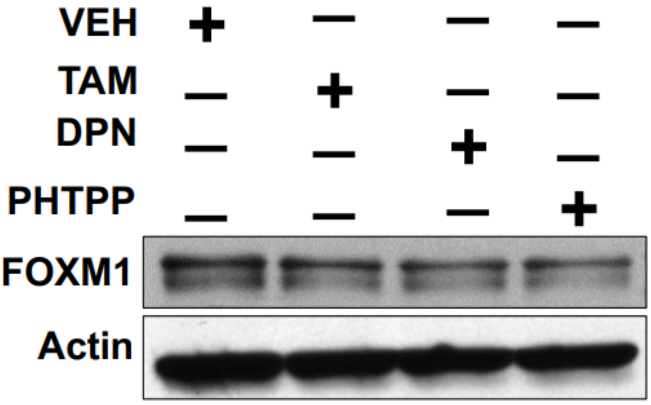

Figure S5. B. Effect of ERβ ligands on FOXM1 expression. OVCAR-3 cells were treated with TAM (2 μM), DPN (10 nM), PHTPP (10 nM) for 24 hrs. Post treatment protein expression of FOXM1 was analyzed by immunoblotting.

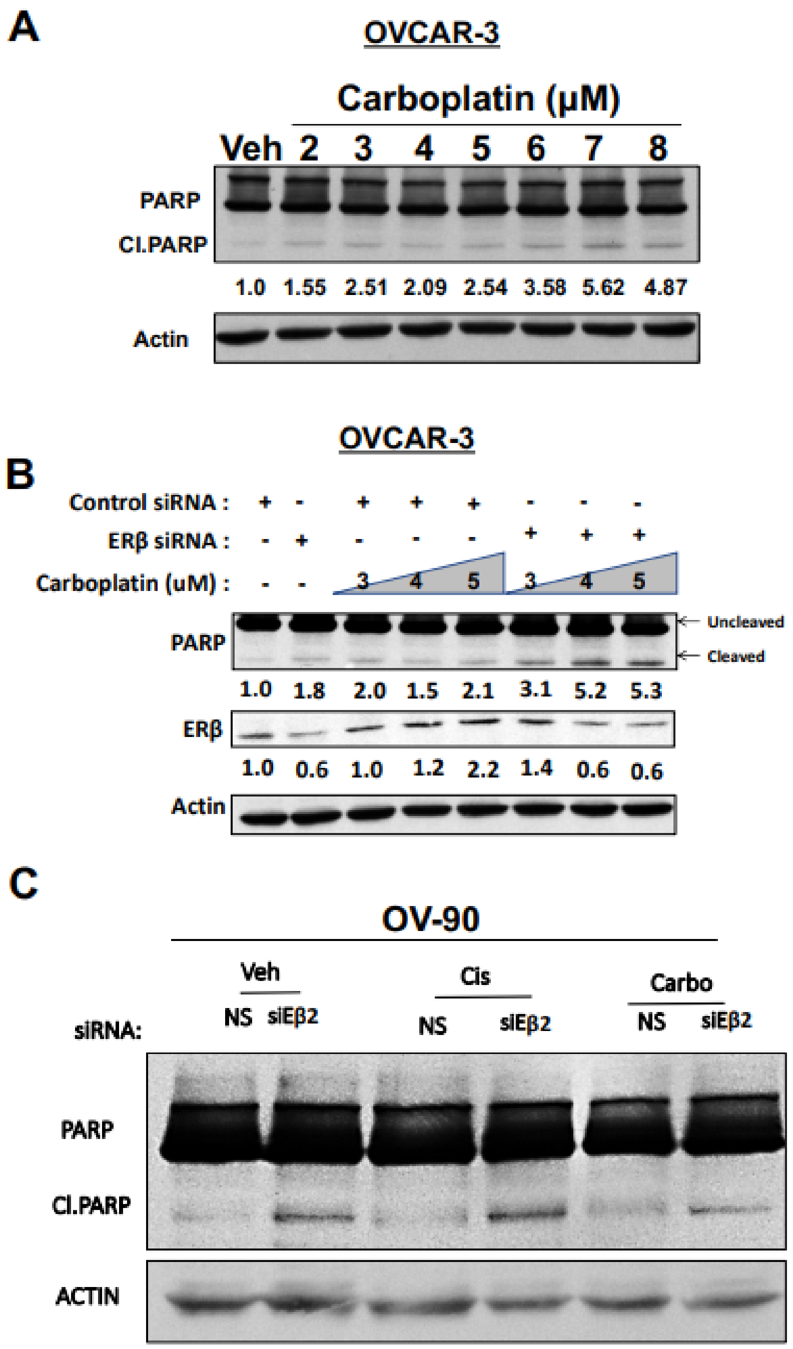

Figure S6. Induction of apoptosis by carboplatin and cisplatin. (**A**) OVCAR3 cells were treated with vehicle and increasing doses of carboplatin (1–6 μM) for 24 hours followed by immunoblotting with PARP antibody. Numbers above the immunoblot bands represent quantitation of respective bands. (**B**) OVCAR3 cells were treated with vehicle and 3 or 4 μM carboplatin for 24 hours followed by immunoblotting with PARP and ERβ2 antibody. Numbers above the immunoblot bands represent quantitation of respective bands. (**C**). Induction of apoptosis with cisplatin (4 μM) and carboplatin (4 μM) in OV90 cells without and with ERβ2 depletion. Expression of cleaved PARP were analyzed by immunoblotting.

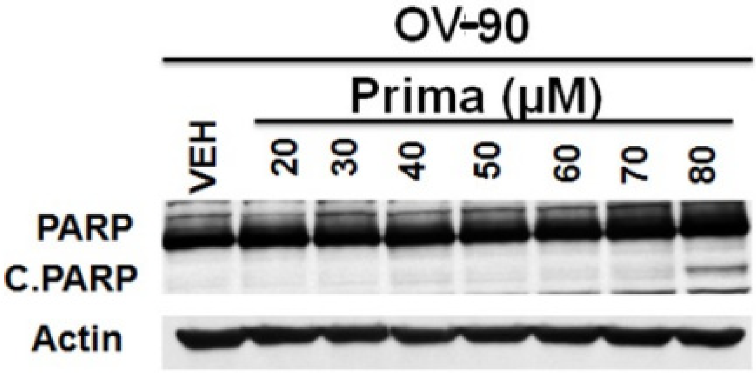

Figure S7. Treatment with PRIMA-1^{MET} induces apoptosis in OV-90 cells. Ov-90 cells were treated with increasing concentration of PRIMA-1^{MET} (20 to 80 μM), followed by PARP-cleavage assay.

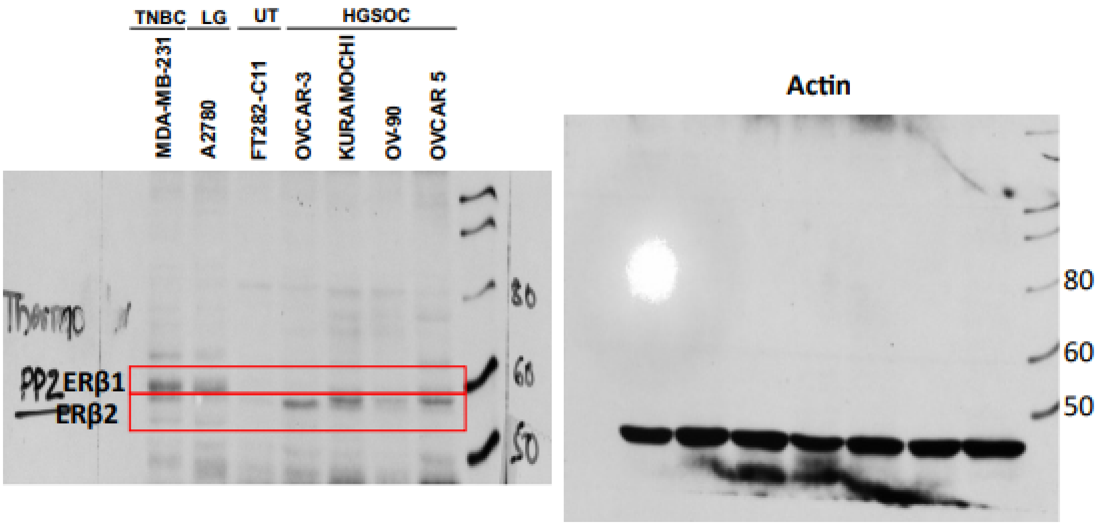

Figure S8. Original blot for Figure. 1B.

Original blot for Fig. 2H

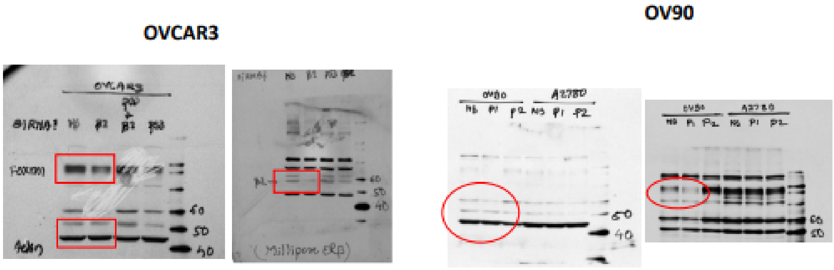

Original blot for Fig. 2I

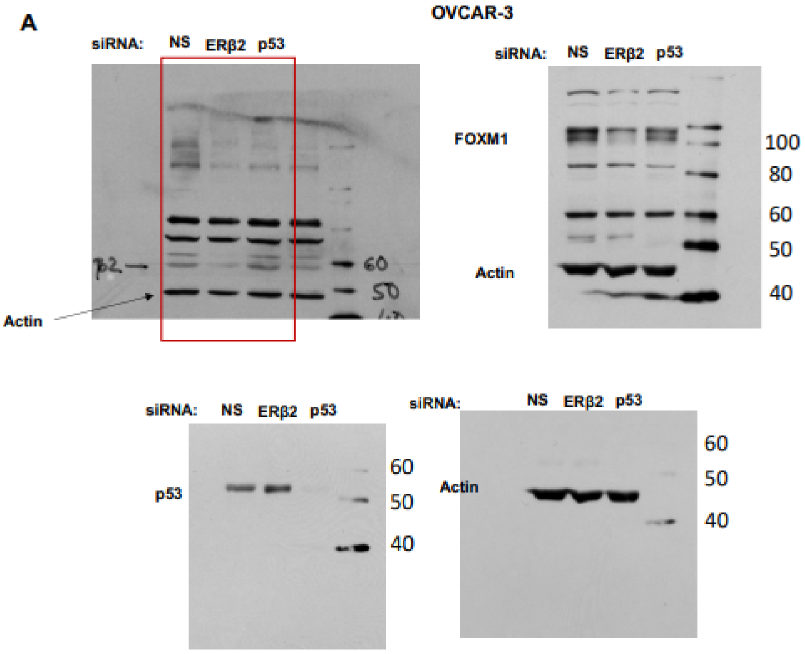

Figure S9.

Original Blots for Figure 3

Figure 3 C

FOXM1

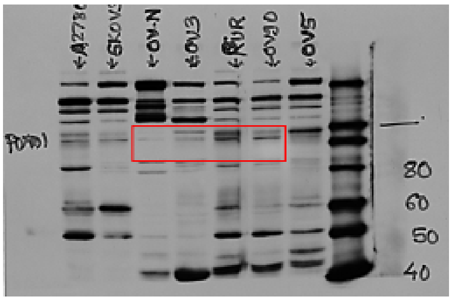

Actin

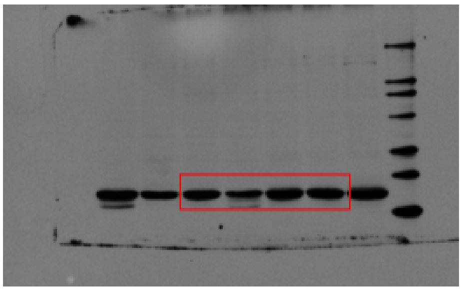

p53

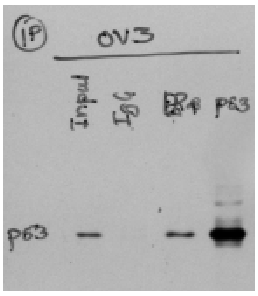

Figure 3 D

ERβ2

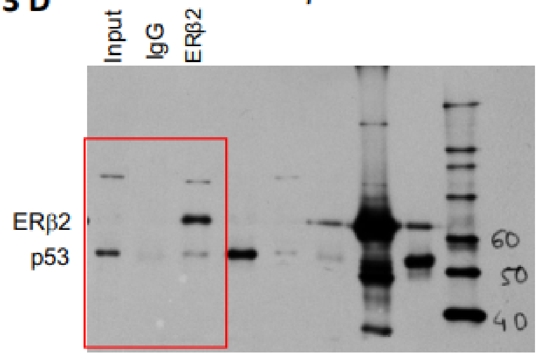

Figure S10.

Original blot for Figure 4A.

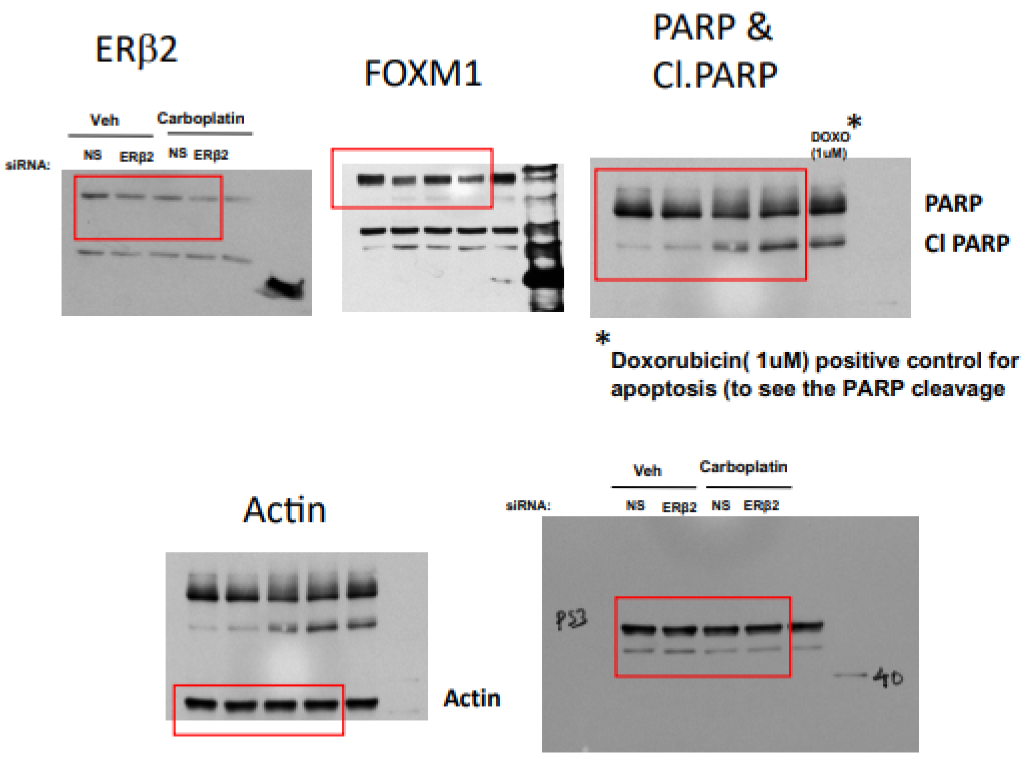

Figure S11.

Original Blot for Figure 5B

Figure 5 B

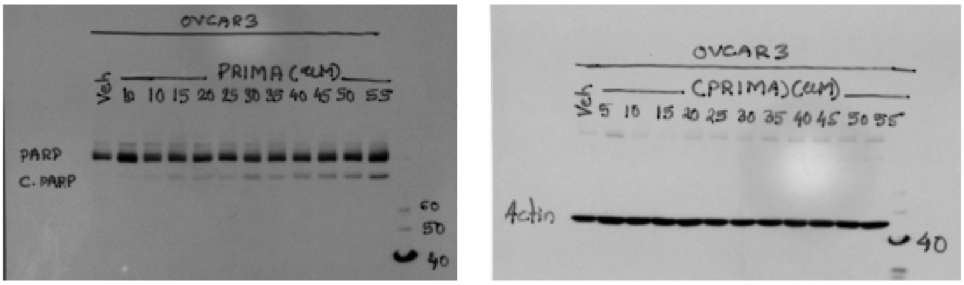

Original Blot for Figure 5C

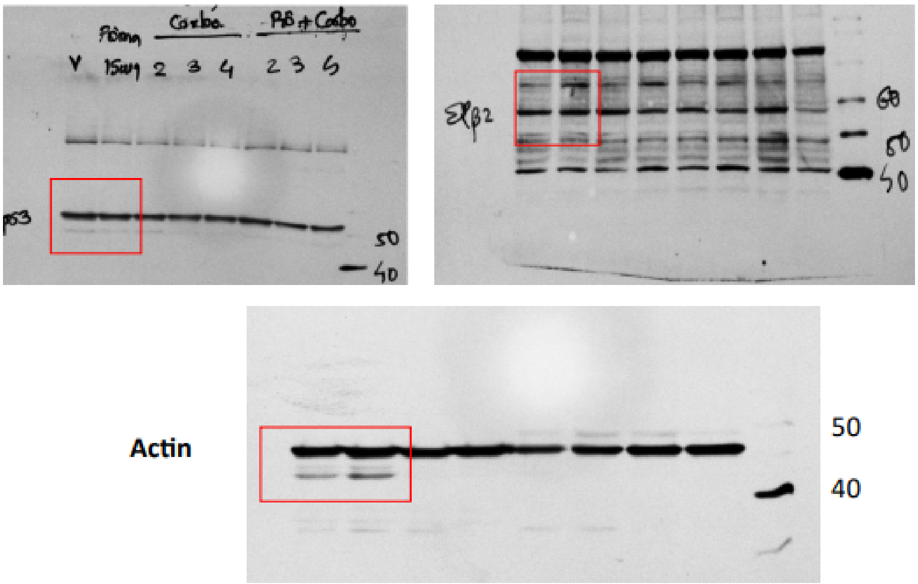

Figure S12.

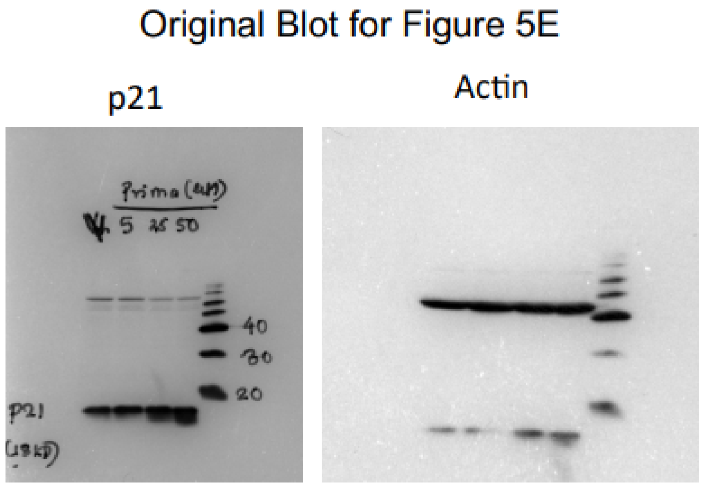

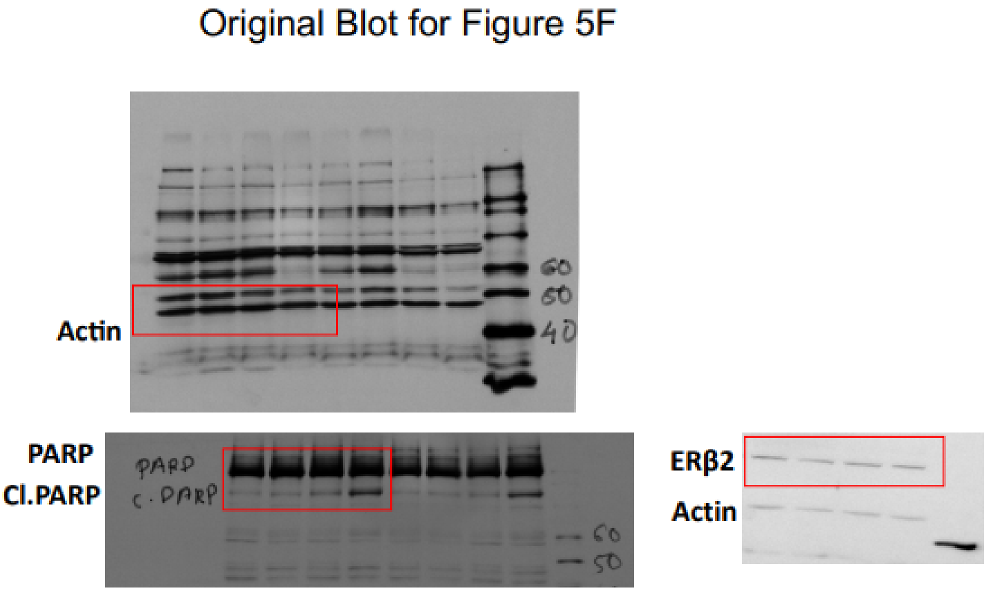

Figure S13.

Table S1. Oligonucleotide primers and siRNAs used in the study.

Quantitative Real time PCR (qRT-PCR) primers		
RT-PCR Primers	ERβ-Forward	5′-CGC CTG GCT AAC CTC CTG ATG-3′
	ERβ -Reverse	5′-GAG CAG ATG TTC CAT GCC CTT G-3′
	ERβ2 - Forward	5′-CGC CGT GAC CGA TGC TTT G-3′
	ERβ2 -Reverse	5′-CCT TTT CTG CCC TCG CA-3′
	FOXM1 - Forward	5′-TGCCCAGATGTGCGCTATTA-3′
	FOXM1 -Reverse	5′-TCAATGCCAGTCTCCCTGGTA-3′
	PLK1-Forward	5′-AAGAGGAGGAAAGCCCTGAC-3′
	PLK1-Reverse	5′-TTCTTCCTCTCCCCGTCATA-3′
	AURKB- Forward	5′-CAGAGAGATCGAAATCCAGGC-3′
	AURKB-Reverse	5′-CCTTGAGCCCTAAGAGCAGAT-3′
	β-Actin-Forward	5′-ATG GGT CAG AAG GATTCC TAT GT-3′
	β-Actin-Reverse	5′-AAG GTC TCA AAC ATG ATCTGG G -3′
	p21-Forward	5′-GAG ACT CTC AGG GTC GAA AAC G-3′
	p21-Reverse	5′-GAT GTA GAG CGG GCC TTT GA-3′
	NOXA- Forward	5′-GCAGAGCTGGAAGTCGAGTGT-3′-3′
	NOXA- Reverse	5′-CTCTTTTGAAGGAGTCCCCTCAT-3′-3′
**ChIP Primers**		
ChIP Primers	Nonspecific -Forward -2965bp	5′- ACACCCTGCCCACTTTCTTA 3′
	Nonspecific- Reverse -2792bp	5′- GATGTTGATACGCATAGGTTTGT -3′
	FOXM1 Forward -2965bp	5′- ACACCCTGCCCACTTTCTTA 3′
	FOXM1 Reverse -2565bp	5′- CTCCAAAGCACAACCAGAGG -3′
**siRNA Sequences**		
siERβ#1	ESR2-HSS103378 ThermoFisher	CCC UGC UGU GAU GAA UUA CAG CAU U
siERβ#2	ESR2-HSS103380, ThermoFisher	CCU UUA GUG GUC CAU CGC CAG UUA U
sip53	sip53- HSS186390, ThermoFisher	GGG ACU UCA ACG AAG GAC ATT

Table S2. Antibodies used in the study.

Protein	Antibodies	Dilutions				Company (Cat. No.)
		IHC	PLA	IB	IP	
**ERβ1**	68-4		-	(1:5000)		Millipore (05-824)
**ERβ2**	Biorad	(1:300)	(1:300)		5μg	Bio-Rad (MCA2279)
**ERα**	HC-20	(1:300)	(1:300)	(1:5000)	5μg	Santa Cruz (HC-20 sc-543)
**p53**	DO1	(1:300)	(1:300)	(1:5000)	5μg	Santa Cruz (SC-126)
	FL393	(1:300)	(1:300)	(1:5000)	5μg	Santa Cruz (SC-6243)
**FOXM1**	Foxm1	(1:100)		(1:5000)		Santa Cruz (C-20)
**p21**	Anti-p21	-		(1:5000)	-	Santa Cruz (SC-397)
**β-actin**	Anti-β-actin	-		(1:5000)		Sigma (A5441)
**PARP**	Anti-PARP	-		(1:5000)	_	Cell Signaling (9542)
**p21**	p21 Waf1/Cip1 (12D1)	-		(1:5000)	_	Cell Signaling (2947)
**Western blot secondary antibodies**	HRP-anti-rabbit	-		(1:10000)	-	Millipore (12-348)
	HRP-anti-mouse	-		(1:10000)	-	Millipore (12-349)
**Immunofluorescence secondary antibodies**	Alexa Fluor 488 anti-mouse	-	(1:100)	_	_	Invitrogen (A11001)
	Alexa Fluor 594 anti-rabbit	-	(1:100)	_	_	Invitrogen (A11012)
**Immunohistochemistry secondary antibodies**	HRP-conjugated anti-rabbit		(1:100)			DAKO (K4003)
	HRP-conjugated anti-mouse	-	(1:100)	_	_	DAKO (PO447)
						Leica (PV-6114)
**IgG negative controls**	Normal mouse IgG	-	(1:100)	1:10000	5μg	Millipore (12-371)
	Normal rabbit IgG	-	(1:100)	1:10000	5μg	Millipore (12-370)
